# A 360‐Degree View of Unprofessional Behaviours Between Nurses and Between Nurses and Medical Colleagues: A Secondary Analysis of a Mixed‐Method Evaluation

**DOI:** 10.1155/jonm/9142351

**Published:** 2026-02-27

**Authors:** Kathleen L. Bagot, Ryan D. McMullan, Johanna I. Westbrook, Ling Li, Tim Badgery-Parker, Rachel Urwin, Sandy Middleton, Elizabeth McInnes

**Affiliations:** ^1^ Nursing Research Institute, St Vincent′s Health Network Sydney, St Vincent′s Hospital Melbourne and Australian Catholic University, Sydney, New South Wales, Australia, svhm.org.au; ^2^ Australian Institute of Health Innovation, Macquarie University, Sydney, New South Wales, Australia, mq.edu.au; ^3^ School of Nursing, Midwifery and Paramedicine, Australian Catholic University, Sydney, New South Wales, Australia, acu.edu.au

## Abstract

**Background:**

Unprofessional behaviour negatively affects staff and patient safety and wellbeing and organisational culture. It typically involves one perpetrator and target/s, as well as staff who may witness, report or respond to the incident, while positive behaviours may buffer experiences. Understanding nurses’ experiences across these roles may support reducing unprofessional behaviour. This is the first 360° view of the roles that nurses play in unprofessional behaviour.

**Aims:**

To examine the frequency, type, severity and impact of unprofessional behaviours between nurses and between nurses and medical personnel; the experiences of reporting and responding unprofessional behaviours; and if nurses acknowledge or exhibit positive behaviours.

**Method:**

Secondary analysis of a mixed‐method study evaluating an all‐staff professional accountability program (*Ethos*) implemented in eight Australian hospitals. Data included (i) cross‐sectional surveys administered pre‐ and postimplementation (longitudinal investigation of negative behaviour surveys: *n* = 5178 baseline [*n* = 2248 nurses] and *n* = 3975 follow‐up [*n* = 637 nurses] surveys), (ii) interviews with middle managers *n* = 30 (*n* = 12 nurses), (iii) 1310 reports of coworker unprofessional behaviours (*n* = 799 submitted by nurses, *n* = 538 about nurses) and 1194 reports of coworker positive behaviours (*n* = 787 by nurses, *n* = 595 about nurses), and (iv) *Ethos* messenger surveys *n* = 60 (*n* = 17 nurses). Analyses undertaken varied by data type: descriptive analysis for quantitative data and content or thematic analysis for qualitative data.

**Results:**

Nurses exhibited unprofessional behaviours (perpetrators), most commonly towards other nurses (62%–90%) and were the targets of nurses (47%–70%) and medical colleagues (4%–34%). Nurses frequently observed unprofessional behaviour, with 51% witnessed it at least weekly. Many (46%) were not comfortable responding, with 44% believing they would not be considered seriously (reporters). Nurses indicated having the skills (83%) and training (87%) to respond to unprofessional incidents. However, they frequently used workarounds (interview theme) or reported insufficient time. Nurses frequently acknowledged others’ positive behaviours (*n* = 1930, 67%), received positive feedback from nurses (1235 behaviours, 83%) and medical colleagues (94 behaviours, 6%).

**Conclusion:**

Nurses’ roles in unprofessional behaviour may include perpetrator, target, observer, reporter, responder and buffer. Individual and organisational‐wide approaches are required to confidently address unprofessional behaviours. Multifaceted culture change programs are needed.

## 1. Introduction

Despite decades of research and evaluation of strategies, nurses’ unprofessional behaviour remains a current concern [1, 2]. Unprofessional behaviours in healthcare have been defined as those categorised as negligence, incompetence or misconduct, as well as other unacceptable behaviours including violent temper outbursts [3]. Such behaviours negatively affect staff and patient safety and wellbeing, and organisational culture [4–7]. Behaviours such as rudeness and bullying have been reported as frequently as weekly [8], with prevalence rates of serious behaviours including harassment ranging between 25% and 59% [4]. Impacts include patient care errors, reduced staff morale [9], turnover intentions and staff losses [10] and a culture of supporting more serious behaviours [8].

As nurses comprise the majority of the healthcare workforce (59%, 27.9 million personnel worldwide) [11], nurses’ involvement in incidents or cultures of unprofessional behaviour is substantial, be it with other nurses or other professional groups. In an ICU setting, nurses and physicians spent most of their time with their same‐discipline colleagues (i.e., nurses with nurses) or with each other; nurses spent approximately 40% of their time with nurses and 12% with physicians, while physicians spent approximately 63% of their time with other physicians and 33% with nurses [12]. As such, nurse–nurse and nurse–physician interactions comprise the majority of clinical personnel interactions. Interprofessional collaboration can improve nurse and physician job satisfaction [13] and patient outcomes [14]. For example, improvements in nurse–nurse and nurse–physician interactions are associated with decreases in hospital‐acquired pressure injuries (31% and 19%, respectively) and inpatient falls (8% and 13%, respectively) [15]. Nurse–physician working relationships form part of nurses’ work environment, and building trust and having mutual respect have been identified as important for strengthening these working relationships [16]. Better work environments and positive interprofessional collaborations are associated with lower patient readmission rates [17]. With a global shortage of nurses [11], identifying and addressing factors that influence recruitment and retention are needed; this includes the experiences and impacts of unprofessional behaviour between nurses, and between nurses and medical colleagues.

When considering unprofessional behaviour, the perpetrator and the target are often the primary focus (Figure 1(a)). Nurses have been identified as both perpetrators [18] and targets [18, 19] of unprofessional behaviour. In a 2016 review of bullying focused specifically on nurses, the authors identified that 20%–25% of nurses experienced bullying [18]. Perpetrators were mainly those in senior positions or long‐term staff, while targets were frequently students and new staff members [18]. Student nurses frequently report being targets of bullying, with up to 58.2% reporting bullying, mainly perpetrated by other nurses (36.8%) [20].

FIGURE 1Six roles nursing personnel may play in unprofessional behaviour. When considering unprofessional behaviour, often the perpetrator and target are the focus (a). However, there are additional roles involved in each incident. There may be others in the vicinity witnessing the incident (i.e., observer role), and those who decide to address the incident‐either by notifying relevant others (i.e., reporter role) and then those who respond (i.e., responder role) (b). At any point in time, an individual from nursing could be behaving as one or more of these six roles in unprofessional behaviour. For example, in one incident, a nurse could be the target and potentially the reporter, while during another incident, they could be the perpetrator and in others, the observer or the responder.

**Figure:**
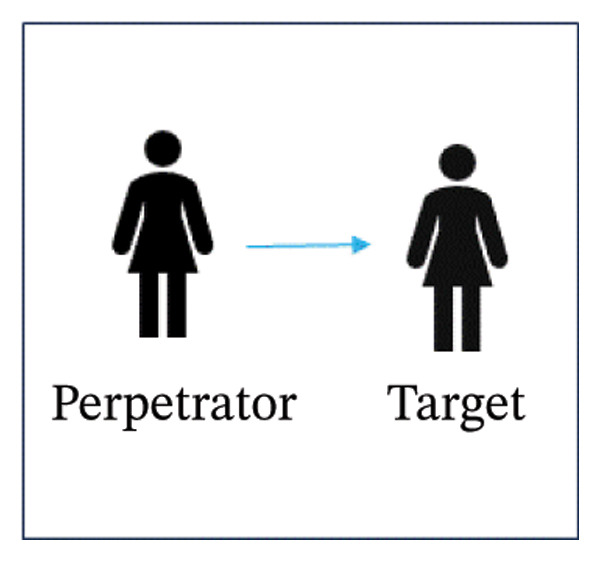
(a)

**Figure:**
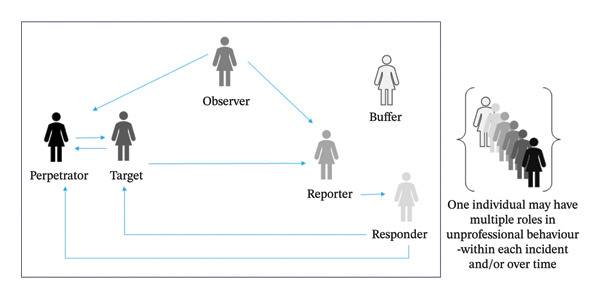
(b)

In addition to those nurses directly involved in incidents of unprofessional behaviour (i.e., as the target or perpetrator), unprofessional incidents also involve those who may be in the vicinity witnessing the incident (observers) and who also experience negative consequences from witnessing unprofessional behaviours (Figure 1(b)). Being a passive bystander to unprofessional behaviour has been negatively related to perceived quality of care and work engagement [10], and witnessing workplace bullying has been associated with turnover intentions [10].

Incidents of unprofessional behaviour may be responded to in the moment (responder) or reported formally or informally to others by the target or by a witness (observer). Once incidents are reported, individuals need to decide if and how to respond to such reports (responder). At any point in time, a nurse could experience being in one or more of these five roles. For example, in one incident, a nurse could be the target and then potentially the reporter, while during another incident, they could be the perpetrator and in others, the observer and potentially the responder.

Literature around these extended roles (i.e., observers, reporters and responders) specific to nurses is limited. A state‐of‐the‐art 2022 review focussing on speaking up (14 review articles with 335 articles, mostly focussing on physicians and nurses, inclusive of students) reported that speaking up was typically defined as raising concerns for patient safety and care in hazardous situations, or raising a concern to someone above them in the organisation’s hierarchy [21]. A 2023 review examining voice and silence (76 studies, 122,009 participants, and 57.8% nurses) concluded that nurses were less likely to express their concerns than doctors or psychologists [22]. Despite its emphasis, the effectiveness of speaking up is unknown [21]. Calls have been made to ensure that those speaking up are heard and responded to appropriately [23]. Such an approach requires exploring not only the experiences of those that speak up (reporters) but also the actions (or not) of those to whom incidents are reported (responders). Those addressing unprofessional behaviour need to consider a comprehensive view of events, including the perspective of the person exhibiting such behaviours (perpetrator), the person who the unprofessional behaviour focuses on (target), anyone who may have witnessed the event (observer), those who report the incident to senior personnel or human resources (reporter) and the person who responds at the time of the event or afterwards (responder).

An additional role to consider is that of those who exhibit behaviours that may buffer against unprofessional incidents. Witnessing positive behaviours and providing or receiving positive feedback may provide individuals with resources to mitigate against the demands associated with negative experiences of unprofessional behaviours [24] (e.g., Job Demands‐Resources model) [25]. In a 2023 mixed‐methods study examining positive feedback of 667 practitioners (*n* = 212 registered nurses, 32%), nurses provided positive feedback significantly more often than physicians or advanced practice providers [26]. While providing and receiving feedback can be complex [27], regular positive feedback from nurse unit managers was associated with nurses’ (*n* = 383) job satisfaction [28]. Positive behaviours by nurses could be encouraged to act as a buffer against the experience of unprofessional behaviours.

### 1.1. The Present Study

No prior study has simultaneously examined all six roles; that is, nurses as perpetrators, targets, observers, reporters, responders and buffers. The *Ethos* program is a professional accountability and culture change program designed to raise awareness about and to address unprofessional behaviours for all staff in healthcare settings [8, 29–35]. Across five participating hospitals, *Ethos* implementation was significantly associated with reduced work‐related bullying, person‐related bullying, physical bullying and sexual harassment and improved capacity for speaking up about unprofessional behaviours [36]. *Ethos* includes capability training and education sessions to enable hospital staff to identify and speak up about unprofessional behaviours, and an online system for reporting positive and unprofessional behaviours. Messages about unprofessional behaviours submitted to the *Ethos* reporting system are triaged, with content delivered by peers (i.e., *Ethos messengers*) or line managers on behalf of the person raising the concern. A multisite, mixed‐methods evaluation was undertaken to explore factors that influenced the *Ethos* program implementation; the reflections of those involved in the *Ethos* program operations, those experiencing or addressing unprofessional behaviours and the use of the online messaging system. The available data from this mixed‐methods study provide an opportunity to explore the various perspectives and experiences of unprofessional behaviour and explore the different roles nurses may play within the same healthcare settings.

The aim of the current study is to examine unprofessional behaviours within nursing and between nurses and medical personnel, including (1) the frequency, type, severity and impact of unprofessional behaviours (nurses as perpetrators or targets of unprofessional behaviour); (2) nurses as witnesses of incidents (nurses as observers) and if observed, if they report them (nurses as reporters), how they respond in the moment or afterwards if incidents are reported to them (nurses as responders) and (3) how nurses support colleagues by providing positive feedback and exhibiting positive behaviours (nurses as buffers), as recognised by other nurses and medical colleagues.

## 2. Method

### 2.1. Design

This study is a secondary analysis [37] of data from the mixed‐methods evaluation of the *Ethos* program implemented across eight Australian hospitals in three Eastern states. As relevant to each aim, the current study draws on full and partial subsets of these evaluation data (i.e., case study framework) [38]. Results from multiple sources are integrated and interpreted in the Discussion section.

### 2.2. Data Sources

To explore the six different roles, six quantitative and qualitative data sources were accessed. Table 1 lists the data sources for each identified role, including time frame, professional group/s providing data, professional group/s being reported on, participant sample size and if any of the data were previously published. Table 2 lists the 26 unprofessional behaviours examined.

**TABLE 1:** Nurses’ contribution to unprofessional behaviour by role, data source, participant reporting and target group, *N*.

Role in nurses’ contribution to UB	Data presented in manuscript or supporting information	*Ethos* project data source	Time frame	Participant professional group^∗^	Focus professional group^∗^	*N*	Some data previously published
Perpetrator	• Table 2: Type • Supporting Table 1: Severity	*Ethos* online Reflection messages	Collected between 2017–2021	All professional groups	Nurses	538 reports (41% of 1310)	(McMullan et al., 2024; Urwin et al., 2023)
	• Supporting Table 1: Demographics • Table 2: type, frequency, target	LION follow‐up survey; type and frequency, target professional group	In last 12 months (between October 2021 and February 2022)	All professional groups	Nurses	782 respondents (55% of 1423 participants)	(Westbrook et al., 2024)

Target	• Supporting Table 2: Demographics • Supporting Table 3: Incivility UB • Supporting Table 4: Extreme UB • Supporting Table 5: All UB • Supporting Tables 6 and 7: By role • Supporting Table 8: Impact	LION Baseline survey Questions UB: ‘has happened to me;’ type, frequency, impact	In last 12 months (between December 2017 and November 2018)	Nurses	Nurses (self)	2248 nurses (46% of 4846 participants)	(Westbrook et al., 2021)
	• Figures 2a and 2b: Role • Supporting Figure 1: Seniority	LION follow‐up survey ‘has experienced in last 12 months;’ type and frequency, perpetrator professional group and seniority	In last 12 months (between October 2021 and February 2022)	Nurses	Nurses (self)	637 nurses (45% of 1423 participants)	(Westbrook et al., 2024)

Observer	• Supporting Table 9: incivility • Supporting Table 10: extreme • Supporting Tables 11 and 12: role	LION Baseline survey questions ‘seen this happen to someone else’	In last 12 months (between December 2017 and November 2018)	Nurses	All professional groups	2248 nurses (46% of 4846 participants)	No

Reporter	• Supporting Table 13	LION follow‐up survey questions ‘comfort with speaking up or reporting UB’ and ‘perceived skills in speaking up’	In last 12 months (between October 2021 and February 2022)	Nurses	All professional groups	637 nurses (45% of 1423 participants)	No
	• Supporting Table 14	Ethos online reporting system Reflection messages	Collected between 2017–2021	Nurses	All professional groups	799 reports by nursing (61% of 1310 reports)	(Urwin et al., 2023)

Responder	N/A	Interviews	Collected between August 2020 and May 2021	Nurses Middle Managers	All professional groups	12 nurses (40% of 30 participants)	(Bagot et al., 2023)
	• Supporting Table 15: Demographics • Supporting Table 16: Views	Ethos Messenger survey	Collected between 16 October and 25 November 2020	Nurses	All professional groups	17 nurses (28% of 60 participants)	(McMullan et al., 2023)

Buffer	• Supporting Table 17	Ethos online reporting system Recognition messages (nurses as providers of positive feedback)	Collected between 2017–2021	Nurses	All professional groups	787 reports by nursing (66% of 1194 reports)	(Urwin et al., 2023)
	• Table 3	Ethos online reporting system Recognition messages (nurses demonstrating positive behaviours)	Collected between 2017–2021	All professional groups	Nurses	595 messages about nursing (50% of 1194 reports)	(Urwin et al., 2023)

*Note:* Incivility = incivility or bullying behaviours (e.g., being ignored or excluded, being spoken to rudely, being given unreasonable workload or deadlines, repeated reminders of errors or mistakes, physically intimidating behaviours, etc.). Extreme = extreme unprofessional behaviours (i.e., physical assault, threats of violence or physical abuse, inappropriate or unwanted touching, demands for sexual favours and sexual assault).

Abbreviation: UB = unprofessional behaviour.

^∗^Professional groups included nursing, medical, allied health, management/administration and nonclinical staff (e.g., food and hotel services).

**TABLE 2:** Frequency of unprofessional behaviours reported about nursing by all professional groups (Nursing as Perpetrator, LION follow‐up survey, *n* = 782).

Behaviour	Target’s role																			
	Nurses				Medical				Allied health and clinical services				Nonclinical services				Management & administrative			
	*n*	%	95% LL^a^	95% UL^a^	*n*	%	95% LL^a^	95% UL^a^	*n*	%	95% LL^a^	95% UL^a^	*n*	%	95% LL^a^	95% UL^a^	*n*	%	95% LL^a^	95% UL^a^
Spoken to rudely (*n* = 492)	304	61.8	57.7	66.1	26	5.3	1.2	9.6	64	13	8.9	17.3	43	8.7	4.7	13	55	11.2	7.1	15.5
Opinions being ignored (*n* = 375)	285	76	72	80.1	16	4.3	0.3	8.3	25	6.7	2.7	10.7	19	5.1	1.1	9.1	30	8	4	12.1
Being given unreasonable workload (*n* = 327)	292	89.3	86.5	92.5	4	1.2	0	4.4	11	3.4	0.6	6.6	10	3.1	0.3	6.3	10	3.1	0.3	6.3
Someone withholding information (*n* = 302)	234	77.5	73.2	81.9	7	2.3	0	6.8	23	7.6	3.3	12.1	15	5	0.7	9.4	23	7.6	3.3	12.1
Being ignored or excluded (*n* = 299)	240	80.3	76.3	84.6	7	2.3	0	6.6	16	5.4	1.3	9.6	14	4.7	0.7	9	22	7.4	3.3	11.7
Shouted at or target of anger (*n* = 234)	168	71.8	66.7	77.5	10	4.3	0	10	16	6.8	1.7	12.6	13	5.6	0.4	11.3	27	11.5	6.4	17.3
Excessive monitoring of work (*n* = 217)	187	86.2	82.5	90.7	5	2.3	0	6.8	9	4.1	0.5	8.7	6	2.8	0	7.3	10	4.6	0.9	9.1
Repeated reminders of errors (*n* = 197)	171	86.8	82.7	91.2	2	1	0	5.4	9	4.6	0.5	8.9	6	3	0	7.4	9	4.6	0.5	8.9
Physically intimidating behaviours (*n* = 116)	88	75.9	69	83.4	5	4.3	0	11.9	8	6.9	0	14.4	1	0.9	0	8.4	14	12.1	5.2	19.6
Being humiliated or ridiculed (*n* = 114)	91	79.8	73.7	87.1	3	2.6	0	9.9	10	8.8	2.6	16	2	1.8	0	9	8	7	0.9	14.3
Hints or signals to quit job (*n* = 108)	91	84.3	78.7	91.1	1	0.9	0	7.8	4	3.7	0	10.5	2	1.9	0	8.7	10	9.3	3.7	16.1
Having responsibility removed (*n* = 98)	86	87.8	82.7	94.1	1	1	0	7.3	4	4.1	0	10.4	1	1	0	7.3	6	6.1	1	12.4
Negative comments about gender, ethnicity, etc. (*n* = 96)	68	70.8	62.5	79.3	9	9.4	1	17.8	9	9.4	1	17.8	2	2.1	0	10.5	8	8.3	0	16.8
Being subject of excessive teasing (*n* = 95)	80	84.2	77.9	90.8	4	4.2	0	10.8	4	4.2	0	10.8	2	2.1	0	8.7	5	5.3	0	11.8
Having unjustified allegations made (*n* = 95)	74	77.9	70.5	85.6	3	3.2	0	10.8	7	7.4	0	15	5	5.3	0	12.9	6	6.3	0	14
Being told sexually explicit or offensive jokes (*n* = 90)	69	76.7	68.9	84.7	5	5.6	0	13.6	6	6.7	0	14.7	2	2.2	0	10.3	8	8.9	1.1	16.9
Treated unfairly because of gender, ethnicity, etc. (*n* = 90)	69	76.7	68.9	84.7	5	5.6	0	13.6	6	6.7	0	14.7	3	3.3	0	11.3	7	7.8	0	15.8
Unwelcome practical jokes (*n* = 61)	53	86.9	80.3	95	1	1.6	0	9.7	2	3.3	0	11.4	1	1.6	0	9.7	4	6.6	0	14.7
Graphic comments (*n* = 51)	42	82.4	74.5	92.8	4	7.8	0	18.3	2	3.9	0	14.4	1	2	0	12.5	2	3.9	0	14.4
Inappropriate or unwanted touching (*n* = 23)	18	78.3	65.2	94.6	0	0	0	16.3	1	4.3	0	20.7	2	8.7	0	25	2	8.7	0	25
Being shown suggestive photo, videos or texts (*n* = 19)	14	73.7	57.9	92.3	2	10.5	0	29.1	1	5.3	0	23.9	1	5.3	0	23.9	1	5.3	0	23.9
Unwelcome sexual flirtations, requests for dates (*n* = 12)	9	75	58.3	100	2	16.7	0	42.6	0	0	0	25.9	0	0	0	25.9	1	8.3	0	34.3
Threats of violence/physical abuse (*n* = 10)	8	80	70	100	0	0	0	28.9	0	0	0	28.9	0	0	0	28.9	2	20	10	48.9
Physical assault (*n* = 5)	4	80	60	100	1	20	0	52.6	0	0	0	32.6	0	0	0	32.6	0	0	0	32.6
Demands for sexual favours (*n* = 2)	1	50	50	100	0	0	0	96.6	0	0	0	96.6	1	50	50	100	0	0	0	96.6
Sexual assault (*n* = 2)	0	0	0	96.6	1	50	50	100	0	0	0	96.6	1	50	50	100	0	0	0	96.6

*Note:* LION = Longitudinal Investigation of Negative Behaviour.

Abbreviations: LL = lower limit, UL = upper limit.

^a^Simultaneous multinomial confidence limits.

Complete methodologies are provided in original publications and details for each role examined in the current study are summarised in Supporting Information. In brief, sources were (i) the all‐staff Longitudinal Investigation Of Negative behaviour (LION) baseline [8] and follow‐up surveys [36], (ii) message content of the all‐staff *Ethos* online system used for reporting unprofessional behaviour and positive coworker behaviours [34, 35], (iii) middle manager interviews exploring how unprofessional behaviour is addressed [29] and (iv) the *Ethos* Messenger survey capturing experiences of those staff delivering messages for reflection [31]. All data were collected after the implementation of *Ethos* at each hospital, with the exception of the baseline survey. All data sources include participants from clinical (i.e., allied health, medical and nursing) and nonclinical (e.g., administration and food services) staff. Analyses were restricted to relevant professional groups depending on the unprofessional behaviour role of interest (see Table 1, columns Participant Professional Group and Focus Professional Group).

### 2.3. Data Analysis

Descriptive statistics (*N*, %, 95 confidence intervals) and chi‐squares were calculated using Excel Version 16, SAS 9.4 and R v4.2. Qualitative data were analysed with NVivo Version 12 [39].

#### 2.3.1. All‐Staff Baseline and Follow‐Up Survey

Unprofessional behaviours were incorporated from the Negative Acts Questionnaire Revised (NAQ‐R) and the Royal Australasian College of Surgeons Discrimination, Bullying and Sexual Harassment Prevalence Survey [40, 41]. These behaviours were categorised into two groups by the research team. Of the 26 unprofessional behaviours listed, 21 were categorised as incivility or bullying (e.g., being ignored or excluded, being spoken to rudely, being given unreasonable workload or deadlines, repeated reminders of errors or mistakes, physically intimidating behaviours, etc.) and five were classified as extreme (i.e., physical assault, threats of violence or physical abuse, inappropriate or unwanted touching, demands for sexual favours and sexual assault). Extreme behaviours were defined as being potentially physical, explicit and criminal.

Frequency counts (*n*) and proportions (*x*/*y*, %) of responses were calculated. The frequency of unprofessional behaviours experienced for incivility/bulling was grouped into three categories: (1) never, (2) occasionally (i.e., around monthly, every few months and 1‐2 times a year) or (3) frequently (i.e., multiple times daily, daily and weekly); and extreme unprofessional behaviours were grouped into two categories: (1) never or (2) ever.

The frequency of incivility/bullying and extreme unprofessional behaviours was stratified by the nursing role type. Nursing roles were categorised as (1) enrolled nurse, (2) graduate nurse or midwife, (3) registered nurse or midwife, (4) Nurse Unit Manager or Associate Nurse Unit Manager and (5) Clinical Nurse Consultant or Specialist or Educator. Responses on five‐point agreement and impact scales were collapsed to disagree (strongly disagree and disagree), neutral (neither disagree nor agree) and agree (strongly agree and agree) and no impact, not sure and negative impact (minor, moderate and major impact).

Analyses performed examined nurses as Perpetrator, Target, Observer and Reporter.

#### 2.3.2. *Ethos* Messenger Survey

Frequency counts (*n*) and proportions (*x*/*y*, %) are reported for participant characteristics and responses to items exploring participants’ perceptions of being an *Ethos* messenger. For responses to open‐ended questions, only those of nurse messengers were included from the inductive analysis originally and reported here. The original analytical procedure included the development of a coding framework using a subset of responses, to identify themes prior to coding all responses. A single analyst undertook an iterative review, with codes and major themes discussed and refined with researchers experienced in the Ethos program and with expertise in qualitative analysis. Illustrative quotes for themes provided by nurses are reported here. These analyses examined nurses as Responder.

#### 2.3.3. All‐Staff *Ethos* Online System Messages

Frequency counts (*n*) and proportions (*x*/*y*, %) of behaviours were calculated by professional groups. The content of each message was analysed using content analysis: unprofessional behaviours were coded deductively to the 26 unprofessional behaviours and positive behaviours to one of seven categories covering positive professional characteristics developed from code of conduct manuals and literature [35]. In addition, content was examined and noted if a patient or staff safety risk was included, or if patient care was impacted. Chi‐squared analyses were conducted to compare severity frequency between professional groups. These analyses were used for nurses as Perpetrator, Reporter and Buffer.

#### 2.3.4. Middle Manager Interviews

Participant demographics were captured verbally including role and years in role. Thematic analysis was undertaken inductively initially within NVivo and then on a virtual whiteboard. A single analyst undertook the recommended 6 analytical phases [42, 43], including interim results being reviewed and probed with those familiar with the interview data and with qualitative analysis expertise. Themes and illustrative quotes specific to nurse middle managers (e.g., Nurse Unit Mangers) as observers and reporters of unprofessional behaviours are provided. These analyses were used for nurses as Observer and Responder.

These results are reported narratively, by each data source, and organised by the following headings: (i) nurses as Perpetrators of; (ii) nurses as Targets of; (iii) nurses as Observers of; (iv) nurses as Reporters of; (v) nurses as Responders to and (vi) nurses as Buffers to unprofessional behaviours.

#### 2.3.5. Ethics Approval

St Vincent’s Hospital Melbourne Ethics Committee (HREC/17/SVHM/237) granted ethics approval. Participants provided informed consent (implied through survey completion, verbal consent prior to interviews), with hospital executive and *Ethos* program lead consent provided for deidentified *Ethos* message data (deidentified for both users and subjects of each submission).

## 3. Results

Nurses included in the mixed‐methods study comprised the largest proportion of staff 44% (*n* = 7875/17769) with medical staff comprising 16% (*n* = 2931). Nurse‐specific data were identified from respondents selecting the nursing option as the professional group (as either reporter or subject). Nurses completed 2248 LION baseline surveys (of 7875 nurses, 29% response rate), 637 LION follow‐up surveys (of 4089 nurses, 16% response rate) and 17 Peer messenger surveys (response rate unknown). Nurses submitted 799/1310 (61%) reports of coworker unprofessional behaviours and 787/1194 (66%) reports of coworker positive behaviours, with 538/1310 (41%) reports of coworker unprofessional behaviour and 595/1194 (50%) positive behaviours submitted about them. There were 12 middle manager interviews with nurses.

### 3.1. Nurses as Perpetrators of Unprofessional Behaviour (Two Data Sources)

#### 3.1.1. Proportion of Nurses Exhibiting Unprofessional Behaviour

Of 1247 follow‐up survey respondents reporting experiencing unprofessional behaviour, 782 (63%) reported experiencing unprofessional behaviour by nurses; see Table 2. Nurses were the majority of respondents (*n* = 508, 65%) with medical staff the fewest (*n* = 37, 5%); see Supporting Table 1.

In total, 538 Reflection messages about nurses’ unprofessional behaviour were submitted, which represents approximately 7% of 7875 nurses. This figure compares with 13% of medical staff (369 reflection messages/2931 medical staff) who received a reflection message from any professional group. Note that one individual could have received more than one message, potentially indicating a lower proportion for each group.

Nurse’s unprofessional behaviour reported by different professional groups via the Ethos online system is presented in Supporting Table 2. The majority of reports about nurses’ unprofessional behaviours were made by other nurses (*n* = 439/538, 81%) and a small proportion by medical staff (*n* = 24/538, 5%).

#### 3.1.2. Type, Target and Severity of Nurses’ Unprofessional Behaviour

Surveys completed could refer to more than one behaviour reported, so total behaviours are greater than the number of surveys. Most frequently reported behaviours exhibited by nurses (Table 2) included being spoken to rudely (*n* = 492 reports; *n* = 304, 62% to nurses; and *n* = 26, 5% to medical), having opinions ignored (*n* = 375 reports; *n* = 285, 76% to nurses; and *n* = 16, 4% to medical), being given unreasonable workload (*n* = 327 reports; *n* = 292, 89% to nurses; and *n* = 4, 1% to medical) and someone withholding information (*n* = 302 reports; *n* = 234, 78% to nurses; and *n* = 7, 2% to medical).

Messages submitted could refer to more than one behaviour, so total behaviours are greater than the number of Reflection messages. In the 538 Reflection messages about nurses, there were 1067 unprofessional behaviours reported (Supporting Table 2). The main behaviours reported about nurses was being spoken to rudely by nurses (*n* = 288 reports; 75%, *n* = 217) were directed towards other nurses, 6% (*n* = 18) to medical staff; being humiliated or ridiculed (153 reports; 80%, *n* = 123) to other nurses, 9% (*n* = 11) to medical; 120 reports of opinions being ignored (79%, *n* = 95) towards other nurses; 104 reports of being shouted at or being the target of anger (79%, *n* = 82) towards other nurses, with 55 reports of being given unreasonable workloads/deadlines or tasks (82% *n* = 67 from other nurses); and 55 reports of having unjustified allegations being made (83% *n* = 66 from other nurses).

The severity of messages submitted about nurses is presented in Supporting Information Table 3. Of those receiving a rating (206/542, 38% not categorised or unactionable), the highest proportion (197/542, 36%) were rated the lowest severity (Level 1) requiring delivery of a reflection by a peer messenger. Level 2 (114/542, 21%) required referral to line manager, and Level 3 (25/542, 5%) required referral to line manager and/or allocation to human resources. There were no Level 4 allocations. Of the 542 reports, 157 (29%) were identified as having a patient safety risk, 220 (41%) as having a staff safety risk and 207 (38%) as impacting patient care.

### 3.2. Nurses as Targets of Unprofessional Behaviour (Two Data Sources)

#### 3.2.1. Frequency of Experiencing Unprofessional Behaviour

Of the 2248 baseline surveys completed by nurses, 2094 (93.1%) reported experiencing at least one unprofessional behaviour in the previous 12 months (Supporting Information Table 4 for all demographics; 85.7% female, 34.6% 25–34 years old, 57.2% registered nurse or midwife and 52.9% employed in nursing over 10 years). Almost all responders experienced incivility or bullying in the preceding 12 months (Supporting Information Table 5); only 3.5% (76/2248) reported never experiencing these types of unprofessional behaviours. Almost 20% (19.8%, *n* = 443/2233) reported experiencing extreme unprofessional behaviours (Supporting Information Table 6).

Frequencies of individual behaviours experienced by nurses are presented in Supporting Information Table 7. Of the incivility/bullying behaviours, being spoken to rudely was most commonly (occasionally or frequently) reported (*n* = 1986/2248, 88%), followed by having opinions ignored (*n* = 1714/2248, 76%) and being given unreasonable workload or deadlines (*n* = 1471/2248, 65%). Behaviours such as being told sexually explicit or offensive jokes/comments at work (*n* = 788/2248, 35%), receiving graphic comments/questions/insinuations about appearance, sexual or private life (*n* = 445/2248, 20%) and unwelcome practical jokes (*n* = 409/2248, 18%) were experienced at least occasionally. Of the extreme behaviours, inappropriate or unwanted touching (*n* = 249/2248, 11%) and threats of violence or physical abuse (*n* = 208/2248, 9%) were most commonly reported. Details of those who were exhibiting these unprofessional behaviours (i.e., perpetrators) were not collected in the baseline survey but are reported from the follow‐up survey in the following.

Approximately 40% of nurses across all roles experienced some form of incivility or bullying behaviour frequently (weekly to multiple times daily), ranging from Clinical Nurse Consultants/Specialists/Educators (*n* = 152/397, 38%) to enrolled nurses (*n* = 69/147, 47%) (Supporting Information Table 8). Across all 21 incivility or bullying behaviours, frequency varied mostly by 10%; that is, regardless of level of role, nurses were reporting experiencing similar frequencies. With extreme unprofessional behaviours, frequency of ever experiencing varied from 15% (*n* = 61/408 Clinical Nurse Consultant/Specialist/Educator) to 23% (*n* = 59/255 Nurse Unit Manager or Associate NUM) (Supporting Information Table 9).

#### 3.2.2. Impacts of Unprofessional Behaviour

Negative impacts most frequently reported were for teamwork (*n* = 1878/2248, 84% of nurses reported unprofessional behaviours had a negative impact on teamwork) and the wellbeing of other staff (*n* = 1791/2248, 81%) and on the participant (*n* = 1737/2248, 78%) reporting the unprofessional behaviour (Supporting Information Table 10). The majority of nurse respondents also reported impacts on patient care (*n* = 1565/2248, 70%) and safety (i.e., frequency of errors or mistakes, *n* = 1610/2248, 73%) and the quality of service provided at their hospital (*n* = 1695/2248, 76%).

#### 3.2.3. Sources of Unprofessional Behaviour

**FIGURE 2:**
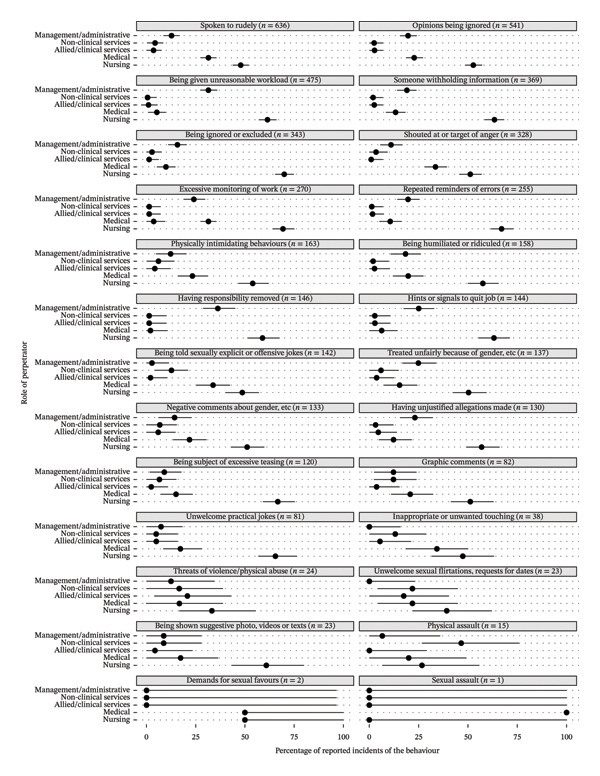
Perpetrators of unprofessional behaviours towards nursing by role (Nurses as Target, LION follow‐up survey; *N* = 637). Note: LION = Longitudinal Investigation of Negative Behaviour; Extreme unprofessional behaviours were physical assault, threats of violence or physical abuse, inappropriate or unwanted touching, demands for sexual favours and sexual assault; remaining 21 behaviours categorised as incivility/bullying.

There were 637 nurses’ responses to the follow‐up survey indicating they had experienced at least one unprofessional behaviour. Which unprofessional behaviour had been experienced and by which professional group are reported in Figure 2. Unprofessional behaviour experienced by nurses often came from other nurses (e.g., 636 reports, *n* = 304 nurses, 47.8% for being spoken to rudely; 270 reports, *n* = 187 nurses, 69.3%, excessive monitoring of work). Unprofessional behaviours experienced by nurses also involved medical personnel, including being spoken to rudely (31.4%, *n* = 200/636), being shouted at or being the target of anger (33.5%, *n* = 110/328) and being told sexually explicit or offensive jokes (33.8%, *n* = 48/142).

The seniority of the staff member exhibiting unprofessional behaviour relative to the target is presented in Supporting Information Figure 1. Across all professional groups, those exhibiting unprofessional behaviours towards nurses were typically more senior. Exceptions were being told sexually explicit or offensive jokes or being the subject of excessive teasing where rates were similar for those senior and at the same level, while of the 24 threats of violence, 12 were more senior, 6 same level and 6 more junior.

### 3.3. Nurses as Observers of Unprofessional Behaviour (One Data Source)

#### 3.3.1. Frequency of Witnessing Unprofessional Behaviour

Almost all (2054/2119, 97%) nurses reported witnessing unprofessional behaviours across all professional groups, with approximately half (1081/2119, 51%) of nurses reporting frequent observations of incivility or bullying and 46% reporting occasional observations (973/2119, 46%, 1‐2 times a year to monthly) (Supporting Information Table 11), with 26% (586/2219) witnessing extreme unprofessional behaviour ever (Supporting Information Table 12).

#### 3.3.2. Roles Within Nursing Witnessing Unprofessional Behaviour

All roles within nursing reported observing incivility or bullying behaviours across all professional groups, with 56% (80/142) of enrolled nurses reporting frequent observations (Supporting Information Table 13). Extreme unprofessional behaviours were also observed by all roles, ranging from 23% (24/104) of graduate nurses or midwives to 32% (82/256) of Nurse Unit Managers or Associate NUMs (Supporting Information Table 14).

### 3.4. Nurses as Reporters of Unprofessional Behaviour (Two Data Sources)

#### 3.4.1. Perspectives on Speaking Up

Nurses’ responses to the follow‐up survey items addressing speaking up (*n* = 637; Supporting Information Table 15) indicated that nurses agreed that speaking up or reporting unprofessional behaviour was important for patient safety (*n* = 553, 93%), knew the proper channels to raise concerns (*n* = 506, 85%), had the skills to speak up if they (*n* = 461, 78%) or others (81%) experienced unprofessional behaviour. However, only about half (*n* = 323, 54%) indicated that they were comfortable speaking up or reporting unprofessional behaviour and that they would be taken seriously if they reported unprofessional behaviour (*n* = 335, 57%). Only one in three nurses (*n* = 197, 33%) agreed that unprofessional behaviour was effectively managed in their hospital, 39% (*n* = 231) agreed that reporting unprofessional behaviour was likely to have a negative impact on their career and 56% (*n* = 334) agreed that they would have support from their supervisor if they reported unprofessional behaviour.

#### 3.4.2. Who Is Reporting Unprofessional Behaviour

Of 1310 Reflection messages submitted across all professional groups, 799 (61% of all messages; a rate of 10 messages per 100 nurses) were submitted by nurses, compared to medical staff who submitted 9% (*n* = 118) of reflection messages (4 per 100 medical staff). Allied health and management and administrative personnel submitting 7% (*n* = 92) of Reflection messages.

#### 3.4.3. Types of Unprofessional Behaviours Reported by Nurses

Within the 799 Reflection messages submitted by nurses, 1583 behaviours were reported (Supporting Information Table 16). The most frequent unprofessional behaviours nurses reported were being spoken to rudely (*n* = 425, 27%), being humiliated or ridiculed (*n* = 217, 14%), opinions being ignored (*n* = 195, 12%) and shouted at or being the target of anger (*n* = 165, 10%). In around half of these instances, nurses were the perpetrators of these behaviours followed by medical staff (e.g., 51% and 32% of being spoken to rudely 57% and 32% of being humiliated or ridiculed, respectively).

When nurses reported somebody withholding information which affects work performance (*n* = 85/1583 behaviours, 5%), incidents were similarly reported about nurses (*n* = 35/85, 41%) and medical (*n* = 36/85, 42%). A substantial proportion of messages submitted by nurses identified the behaviour as posing a patient safety risk (*n* = 273/799, 34%), staff safety risk (*n* = 323/799, 40%) and/or as impacting patient care (*n* = 376/799, 48%).

#### 3.4.4. Response of Perpetrator When Concern Raised

When submitting messages, nurses reported the perpetrator’s response almost half the time (*n* = 350/795, 44%). Perpetrators responded generally in two ways: that is, by either calling out the person who was speaking up to them (reprimand, challenge, command, defend *n* = 96/298, 32%) or by being reflective (summarise, empathize, enquire, *n* = 81/298, 27%) about their behaviour.

### 3.5. Nurses as Responders to Unprofessional Behaviour (Two Data Sources)

#### 3.5.1. Nurses’ Process of Responding When Concerns Were Reported to Them

Nurse middle managers’ interviews (*n* = 12) indicated they were generally comfortable addressing unprofessional behaviours; incidents that they witnessed or those that were raised with them by staff. They indicated they assessed each complaint to determine if it was genuine and took action depending upon aspects of the incident, including preferences of the person raising the concern, the behaviour (e.g., type, frequency and impact) and the individual committing the behaviour (e.g., if have good working relationship and if known to be a repeat offender). Middle managers sometimes addressed the concern directly (i.e., speaking with perpetrator and encouraging person raising concern to address) or indirectly (e.g., advise person raising concern to avoid person committing unprofessional behaviour and changing roster so target not working with perpetrator). At times, they took no action and justified unprofessional behaviours:

“I guess it’s a very stressful environment … in the middle of a case, if things don’t go right, the [professional] behaviours will go out the window. … But you can see there’s a life on the line and it’s not black and white.” (Participant 017)

“I actually think it’s more about individual personalities. … there’s one in particular [specialist] at the moment that’s behaving quite poorly to a lot of different people, to people that are in senior roles and junior roles. But that’s his personality that’s driving that.” (Participant 009)

Many noted additional workload and workarounds that were required:

“I have performance managed people around professionalism before. … They are very difficult to get to identify it [unprofessional behaviour] as an issue and then get the change that’s required. Then on top of that, it’s really lengthy, it takes a long, long time.” (Participant 005)

Despite preferring to address unprofessional behaviours, this came with a personal toll:

“I know this person’s very challenging and that they might push back, so I just need to take a moment to really think about the conversation I′m going to have, and how I’m going to have it, so that I do it in a way that hopefully doesn’t escalate it in a negative way.” (Participant 002)

#### 3.5.2. Nurses’ Experiences Delivering Feedback to Those Demonstrating Unprofessional Behaviour

There were 17 *Ethos* Messenger surveys completed by nurses, with 15 currently acting in the role of messenger (Supporting Information Table 17). Participants had a range of experience as a messenger, being in the role for between less than 3 months (*n* = 1, 6%) to over 12 months (*n* = 7, 41%). While two (12%) had yet to deliver a message, two (*n* = 2, 12%) had delivered 6–10 messages, with the majority delivering 1–5 messages (*n* = 11, 65%). Reasons for becoming an *Ethos* messenger included categories of belief in the philosophy of the *Ethos* program (e.g., “Liked the sound of the program and thought I could make a difference to the culture of the hospital.” Participant 12), to contribute to a positive hospital culture (e.g., “I like that it represents the hospital values. It reinforces how we should talk and interact with staff.” Participant 10) and individual personal reasons (e.g., “Opportunity to actively participate in creating a culture where it is okay to speak up and where unprofessional behaviour is addressed through reflective conversation. It was also a professional development opportunity.” Participant 9) and because they had been approached (e.g., “I agreed because I felt that staff need a way they could speak up.” Participant 4).

Responses to questions about their experience as *Ethos* messengers are presented in Supporting Information Table 18. Nearly all agreed that they had the skills to be successful in the *Ethos* messenger role (*n* = 14/15, 93%), were satisfied with the training (*n* = 13/15, 87%) and had sufficient support to fulfil the role (*n* = 13/15, 87%). Only approximately half felt they had sufficient time to carry out the role effectively (*n* = 8/15, 53%), felt valued in their role (*n* = 8/15, 53%) and that hospital management were committed to the success of the *Ethos* program (*n* = 9/15, 60%).

### 3.6. Nurses as Buffers of Unprofessional Behaviour (One Data Source)

#### 3.6.1. Nurses Reporting Positive Behaviours of Others

There were 1195 Recognition messages submitted in the *Ethos* online system, of which 787 were submitted by nurses, providing the highest number of Recognition messages by staff. Accounting for professional group size, nurses were the greatest contributor of Recognition submissions, with 9.9 submissions per 100 nurses (*n* = 7875 nurses), whereas there were 3.8 submissions per 100 medical staff (*n* = 99).

The majority of submissions by nurses were about other nurses (*n* = 493/787, 63%), followed by messages about medical staff (*n* = 126/787, 16%). Of 2872 total positive behaviours reported, 1930 (67%) were reported by nurses (Supporting Information Table 19). The most frequently reported behaviours were notable nontechnical skills (625/1930, 32%; e.g., teamwork, communication and collaboration), positive values‐driven behaviour (579/1930 30%; e.g., ethics, compassion, empathy and support) and patient care delivery and enhanced performance (425/1930 22%; e.g., treatment, safety and quality of care).

#### 3.6.2. Nurses Exhibiting Positive Behaviours Reported by Others

In total, hospital staff made 593 Recognition messages about nurses, with the majority submitted by nurses (*n* = 493, 83%), medical (39/593, 7%) and allied health and clinical services (37/593, 6%). Accounting for professional group size, nurses remained the highest contributor of Recognition submissions, with 7.6 submissions per 100 nurses (*n* = 7875 nurses), followed by medical (*n* = 202 messages of 2931 medical staff, 6.9 submissions per 100 staff).

There were 1483 positive behaviours reported about nurses in the 593 Recognition messages (see Table 3). The most frequently reported were notable nontechnical skills (466/1483, 31%), positive values‐driven behaviour (444/1483, 30%) and patient care delivery and performance enhanced (330/1483, 22%).

**TABLE 3:** Frequency of positive professional behaviours reported about nursing by professional groups (Nursing as Buffer, *Ethos* online reporting system, Recognition messages, *n* = 595).

Behaviour reported in recognition messages about nursing (*n* = 1483 positive behaviours reported)	Total	Nursing		Medical		Allied health		Other clinical		Nonclinical	
		*N*	%	*N*	%	*N*	%	*N*	%	*N*	%
Patient care delivery and performance enhanced (treatment and care effectiveness, safety, manner and quality of care, patient‐centredness and timeliness)	**330**	269	82	25	8	18	5	5	2	13	4
Notable nontechnical skills demonstrated (teamwork, coordination, collaboration, communication, other)	**466**	385	83	34	7	20	4	11	2	16	3
Notable technical skills demonstrated	**11**	9	82	1	9	1	9	0	0	0	0
Overcompensating for gaps in resources, task, skill shortages through personal effort and time	**48**	40	83	3	6	1	2	3	6	1	2
Positive values‐driven behaviour demonstrated (ethical, compassionate, empathetic, supportive, respectful, affording others dignity)	**444**	370	83	26	6	23	5	8	2	17	4
Advocacy for other people demonstrated	**37**	27	73	3	8	4	11	2	5	1	3
Positive leadership, teaching or coaching skills demonstrated	**147**	135	92	2	1	3	2	2	1	5	3
Total	**1483**	1235	83	94	6	70	5	31	2	53	4

*Note:* The total column is the number of messages relating to the positive professional behaviour reported. For example, 330 = 330 behaviours were about patient care delivery and performance enhanced. The column can be headed total N.

## 4. Discussion

This is the first study to examine the six different roles that nurses play in unprofessional behaviour in healthcare settings, drawing from multiple data sources from a multisite mixed‐methods study. We examined nurses as perpetrators or targets of unprofessional behaviour (aim 1), nurses as observers, reporters and responders (aim 2) and nurses as buffers (aim 3). Using a convergence approach, we combine results from the secondary analyses (see Table 4) through weaving [38] and present a narrative integration.

**TABLE 4:** Summary of nurses’ roles in unprofessional behaviour.

Role in relation to unprofessional behaviour	Quantitative result from LION baseline, LION follow‐up and *Ethos* peer messenger surveys and *Ethos* program online message system	Qualitative example from *Ethos* program online message system and interview data
Perpetrator	63% (782/1247) of all participants reported experiencing unprofessional behaviour by a nurse in the previous 12 months	‘Following the bed meeting, while other NUMs were present, [name deleted] approached me and demanded to know why I couldn’t move her medical patient in an aggressive manner. I told [name deleted] there were 5 unallocated medical patients in ED and 5 to come out of ICU, 2 of the ED patients had been waiting 3 and 4 days. She dismissed my explanation and just walked off.’ (Reflection message submitted by a nurse about another nurse)
Target	93% (2094/2248) of nurse participants reported experiencing at least one unprofessional behaviour in the previous 12 months	‘[Name deleted]’s response to me when I said I was drawing up the local as he asked for was “Oh she’s so silly. The first thing she asked me was what was the local I wanted and she hasn’t even bothered to get it ready yet. She couldn’t even remember to do that.” This was said in a very dismissive and demeaning tone. It was also only the first of many similar comments.’ (Reflection message submitted by a nurse about medical)
Observer	97% (2054/2119) of nurse participants reported witnessing unprofessional behaviours	‘I have personally observed [name deleted] be dismissive of nursing staff concerns and have had multiple other staff discuss his poor demeanour and level of responsiveness to nursing concerns.’ (Reflection message submitted by a nurse about medical)
Reporter	54% (323/637) of nurse participants reported being comfortable speaking up or reporting unprofessional behaviour	‘I want the above workplace behaviour to stop. However, my greatest fear is that by reporting the above behaviour, there is just going to be a terrible backlash against me for reporting it.’ (Reflection Message submitted by a nurse about a nurse)
Responder	87% (13/15) of nurse Ethos Peer Messengers were satisfied with the training	“No one will ever put it in writing. … He [specialist] is a bully … your old‐fashioned, wealthy, tall, big man … we roster so that it works out okay.” (Participant 017, middle manager nurse interview)
Buffer	67% (1930/2872) of total positive behaviours were reported by nurses Nurses were the greatest contributor of Recognition messages (9.9 submissions per 100 nurses compared to 3.8 submissions per 100 medical staff)	‘During an extremely busy Operating day and a shortage of staff, resources and skill mix, [name deleted] took responsibility of a General operating list. She organised and communicated with her colleagues well, was thoughtful and kind. She did not have any breaks and offered to stay past her shift. It was an absolute privilege to work with [name deleted]. (Recognition message submitted by a nurse about a nurse) [Name deleted] supported me and asked if I was OK after being told off by the registrar this morning and to not take it personally as my second day of internship. I really appreciated that she cared about my wellbeing after the incident, and that the nurses are here to also care for us.’ (Recognition submitted by medical about a nurse)

Nurses exhibited a range of unprofessional behaviours, most commonly towards other nurses, and were also the targets of other professional groups, typically by those more senior than them and by medical professionals. Almost all nurses across all levels of seniority reported experiencing incivility or bullying behaviours in the previous 12 months. Nurses frequently observed incivility or bullying, with approximately one in four witnessing extreme unprofessional behaviour. Although the impacts on wellbeing, teamwork and safety were identified, substantial barriers mitigated against reporting unprofessional behaviour, including the time and effort required, and perceived potential negative career consequences. Those responding to reports of unprofessional behaviours had the skills although only some had received specific training, and frequent workarounds were in place to avoid directly addressing perpetrators. Conversely, nurses provided positive feedback to professional groups (nursing, medical and others) and also received recognition for their own positive behaviours from others.

Our results show that nurses are active in each of these six roles: nurses are the perpetrator, target, observer, reporter, responder and buffer to unprofessional behaviour. These roles were evidenced within nursing (intragroup unprofessional behaviours) and between nurses and medical (intergroup unprofessional behaviours). As such, this work provides insights into these working relationships and by applying this nurses’ lens, we can identify additional strategies to address and reduce such incidents.

### 4.1. Intragroup Unprofessional Behaviours

#### 4.1.1. Nurses as Perpetrators and Targets (Aim 1)

Consistent with the literature [20], nurses reported experiencing unprofessional behaviours from other nurses (nurses as perpetrators and targets), mainly from those more senior. Behaviours included being spoken to rudely, being humiliated or ridiculed, having opinions being ignored or being the target of anger. While most *Ethos* messages only required a peer conversation, over one‐third indicated a serious event or pattern of behaviour. Incivility between nurses (lateral violence) [9] has been reported early in nurses’ careers, including during clinical placements as part of nurses’ education [1]. A 2023 review of 421 studies focussing on student nurses’ (*n* = 14,984) experiences of workplace violence (e.g., racism, bullying, physical or sexual aggression) concluded that violence during nurses’ placements is common [20]. These early experiences across the spectrum of unprofessional behaviour contribute to expectations of nursing workplace culture, and subsequent acceptance of being the targets, and likely once senior, potentially subsequent perpetrators of unprofessional behaviour. Experiencing such behaviours may even be considered a rite of passage [44], perpetuating workplace culture depicted in the phrase ‘nurses eat their young’ [45]. This culture of accepting unprofessional behaviour extends to the experiences of others witnessing unprofessional behaviour and responses by those in positions to address it.

#### 4.1.2. Nurses as Observers, Reporters or Responders (Aim 2)

Nurses reported frequent observations of unprofessional behaviours and of enduring the emotional labour of witnessing behaviours [46], regardless of whether they act or not. Despite reporting having the skills, nurses were generally not comfortable speaking up. Concerns were expressed that they would not be taken seriously, the hospital would not manage the concern effectively and that there would be negative impacts on their career. If observers do not act, this provides passive support for negative behaviours to continue [18], further contributing to a culture of acceptance. Previous work has identified that nurses did not speak up as bullying was perceived as inevitable, as part of the nurse’s role, and due to the potential consequences of doing so [47]. For those witnessing incidences, passive bystanders’ behaviours are negatively related to the perceived quality of care (predicts nurse‐assessed patient safety such as medication errors and fall injuries) and work engagement [10]. When unprofessional behaviour is considered a normative experience, this precludes responders acting. If unprofessional behaviours are considered part of the workplace culture, nurses as targets, observers, reporters and responders are less likely to address incidences of unprofessional behaviour, despite reporting having the skills to do so.

### 4.2. Intergroup Unprofessional Behaviours—Nurses and Medical

Nurses were also both perpetrators and targets of other groups’ unprofessional behaviours. In particular, while nurses and medical personnel exhibited unprofessional behaviours towards each other, it was more common for medical staff to exhibit these towards nurses. Most unprofessional behaviours exhibited were behaviours relating to communication (e.g., spoken to rudely) or physical behaviours (e.g., physically intimidating behaviours). Stronger interprofessional collaboration and positive work environments have been identified to improve patient care and safety (e.g., reduced pressure ulcers and patient falls) [14, 15] and patient outcomes (e.g., lower patient readmission rates) [17]. A 2015 meta‐analytic review of 51 surveys (13,132 nurses and nursing students and 5650 physicians and medical students) revealed that nurses reported more positive attitudes towards collaboration than physicians, and compared with physicians, nurses reported weaker current collaborative practices [48]. The findings of our analysis (e.g., both nurses and medical provided and received positive feedback from each other) and this review suggest that despite nurses being the targets of medical staff unprofessional behaviour, they acknowledge and are open to positive interprofessional working relationships. Improvements in interdisciplinary collaboration helps build trust and respect, reduce the influence of long‐established perceived hierarchies between nurses and medical [49] and increase nurse and physician job satisfaction [13].

### 4.3. Nurses as Buffers (Aim 3)

Nurses provided and received the highest rates of positive messages from across professional groups. Positive feedback has been suggested as part of culture change [50], with reflecting and rewarding positive behaviours proposed to address lateral violence [2], as a contribution to safety models [51], and to improve workplace culture and morale [52]. Positive feedback has also been associated with intrinsic motivation and learning, motor skills, self‐efficacy and as such, may improve performance [51, 53]. Our results suggest that although nurses are perpetrators of unprofessional behaviours, and may not speak up when experiencing or witnessing incidences, they contribute positively to workplace culture through providing positive feedback to other nurses and colleagues.

### 4.4. Healthcare Workplace Culture

Nurses may contribute to a tacit acceptance of a workplace culture of unprofessional behaviour as a perpetrator, by not reporting or responding in the moment as the target or observer or not directly responding when incidents are reported to them. Nurses’ experience of unprofessional behaviour begins during their undergraduate education [20] and continues throughout nurses’ work lives [47]. A 2021 qualitative review found one thematic response to bullying was ‘surrendering to the culture of bullying’ (p. 4315) [47]. Our results showed that only approximately half of nurse survey respondents thought their supervisor would be supportive, and even fewer (1 in 3) reported the hospital as effective in managing unprofessional behaviour. While some nurses had received specific and useful training to deliver feedback on individuals’ reported behaviour (as *Ethos* peer messengers) [29], such upskilling support was limited more generally. Middle managers noted preferences for addressing issues directly, but reported that this resulted in a personal and professional toll. In turn, those addressing unprofessional behaviours reported passive approaches (e.g., changing rosters), rather than active approaches such as directly addressing the perpetrator or escalating to human resources or senior management. The use of workarounds (e.g., changing rosters and recruiting specific personalities) may mitigate an incident but perpetuates that influential perpetrators are supported, and unprofessional behaviour can continue. Inappropriate communication may be explained away by stressors or ‘just a joke’ but have detrimental impacts, undermining the values of the healthcare and workplace safety. Our results suggest that broader factors such as individuals prioritising the importance of maintaining collaborative working relationships over addressing unprofessional behaviour [46] and a leadership and management workplace culture [32] of justifying or accepting individuals’ unprofessional behaviours (e.g., known repeat offenders) influence reporting and responding to unprofessional behaviours.

Our findings suggest that ‘speaking up’ interventions that focus primarily on nurses who have experienced or witnessed unprofessional behaviours places the responsibility on the target or observer (who may be experiencing distress). These interventions are further limited if subsequent individual or organisational processes do not support addressing perpetrators’ behaviours [23, 54]. In addition, unprofessional behaviours are commonly reported to be exhibited by those more senior than the target. Therefore, if incidents are reported to supervisors, it is likely that it will be the peers of perpetrators who are called on to address the incident. The entrenchment of power dynamics and hierarchies and justifications that preserve negative workplace cultures contribute to the perpetuation of unprofessional behaviours in healthcare settings [2, 29]. Immediate supervisors, managers and executive management need to actively support those raising concerns and directly address those exhibiting unprofessional behaviours. Also required are systems‐based processes that emphasise professional codes of ethical behaviour, organisational policies and procedures that illustrate that unprofessional behaviour is not tolerated and that reporting or responding to unprofessional behaviour will not have negative repercussions. While we have focused on nurses, organisational‐wide programs are required to ensure hospital‐wide culture change, regardless of professional group.

When low‐level unprofessional behaviour (e.g., rudeness) is not addressed, this may lead to more serious behaviours [8] and a culture of acceptance [20]. Conversely, although not specific to nursing, addressing low‐level behaviours can reduce future incidences of unprofessional behaviour [55]. One study that evaluated a peer review system (Coworker Reporting System; CORS) found 71% of physicians and advanced practitioners who received a report regarding unprofessional behaviours had no subsequent reports in the following year. The *Ethos* program demonstrated significant reductions in experiencing extreme unprofessional behaviour (32% overall reduction in odds of experiencing). These results suggest that interventions supporting reporting and feedback to perpetrators of incidences, in conjunction with training on how to identify unprofessional behaviour, can be successful in changing behaviours.

Nurse retention, particularly in the current climate, is critical. Nurse turnover has been associated with reduced patient satisfaction, higher patient falls, pressure ulcers and medical errors [56], as well as reduced mental health and job satisfaction for nurses, and workload stress [56]. Due to the scale of the nursing workforce, changes in the experiences of a small proportion of nurses could make a substantial difference to overall workplace culture, increasing job satisfaction and retention of nurses in the workplace.

### 4.5. Recommendations

This 360° view illustrates that interventions only tailored for individuals to speak up when experiencing or witnessing unprofessional behaviour are insufficient in addressing embedded behaviours. First, each nurse may experience one or more roles—target, observer, reporter and responder—involved in a single incident or multiple incidents over time. Interventions that only focus on the target speaking up are inadequate. Developing or selecting potential interventions should include components to address all identified roles. Second, nurses must have the skills to not only identify and report if they are a target or observer but also the skills and support to actively and directly respond when reports are made to them. Such skills are relevant for all nurses, from students undertaking clinical placements, to management and executive‐level nurses, but training may be tailored to role and be accessible to all. Interventions should include education and training alongside opportunities to practice new skills [9] that identify and address unprofessional behaviour, including how to deliver, receive and reflect on constructive feedback respectfully. Identifying when perpetrators’ behaviours are being justified or indirectly addressed is also warranted. Third, systemic processes [2] must be in place to support all roles involved in unprofessional incidents. Systems should include mechanisms to easily report incidents, provide feedback confidentially and monitor incident frequency by individual over time [30]. The use and availability of reporting systems should be included in training (e.g., from orientation to annual refresher programs) to illustrate the importance of improving workforce experiences and workplace culture. Fourth, procedures and policies drawing on professional ethics and institutional values (not just one individual’s expectations) to ensure that unprofessional behaviour, regardless of perpetrator’s role or seniority, is not tolerated. Adjacent programs, such as mentoring programs [57, 58] must promote appropriate behaviours, not perpetuate justifications or support workarounds. Fifth, interventions should also incorporate the identification and reporting of positive professional behaviours. This inclusion ensures positive behaviours are encouraged and recognised, supporting culture change [59] and potentially buffering unprofessional behaviours. To illustrate the value, those receiving positive feedback may have it documented and included in their personnel files [51]. Sixth, when implementing culture change interventions, identifying and addressing local barriers (e.g., readiness of personnel and communication about program) and incorporating facilitators (e.g., champions and leadership demonstrating action) should also be considered [33]. While based on data from five Australian hospitals, our results are in line with the international literature (cited above). In turn, these recommendations are likely relevant to other large western health settings.

### 4.6. Strengths, Limitations and Future Research

This is the first exploration of the multiple roles within unprofessional behaviour, focussing on nurse–nurse and nurse–medical relationships. Strengths include the mixed‐method approach, conducted within multiple settings providing insights of six different roles contributing to addressing unprofessional behaviours within the nursing profession and between nurses and medical. Despite the strengths, a number of limitations are to be considered. Anonymous survey reporting and deidentified data may offset some of the impact of self‐report data and social desirability bias. As with all secondary analyses, the key limitation is that these data were not collected specifically to identify or explore the six different roles that we present. While some data sources were applicable across all staff professional groups (e.g., Longitudinal Investigation Of Negative behaviour baseline and follow‐up surveys), others were limited to specific groups (e.g., responders were limited to middle managers and *Ethos* peer messengers and not team leaders, senior managers or human resources), time periods varied (refer Table 1) and each data source has its own limitations, as detailed in previous publications. The mixed‐method data, however, does offset some of these single data source limitations and provides important insights and avenues for future research. For example, exploring the impacts more deeply (e.g., how is teamwork affected) or how positive behaviours (e.g., receiving positive feedback) may mitigate (or not) the experience of unprofessional behaviours would be particularly informative. While some of the recommendations may be relevant to all healthcare settings, exploring how unprofessional behaviour may vary within hospital settings (e.g., different wards) or other health settings (e.g., General Practice) may elicit different or nuanced recommendations. The study was undertaken during the COVID‐19 pandemic associated with an increase in personal and professional demands. However, there was little support for COVID‐19 being a catalyst for a widespread increase in unprofessional behaviour [60].

## 5. Conclusions

We present the first 360‐degree view of nurses’ roles in relation to unprofessional behaviour, addressing six key roles: perpetrator, target, observer, reporter, responder and buffer. Addressing both individual and organisational factors is required to address the culture of accepting unprofessional behaviours by nurses, and to nurses. Interventions need to extend from focussing on targets speaking up to include training in how to identify, report and respond to incidents, with hospital‐wide policies and procedures implemented to support all roles involved in each incident. Given the concerns regarding recruitment and retention of the nursing workforce, and patient and staff safety, it is incumbent on health services to urgently address the reasons for the perpetuation of a workplace culture that appears to normalise unprofessional behaviours and workarounds. Ensuring that the clinical workplace is conducive to best clinical practice, nurse and patient safety, nurses’ job satisfaction and their health and wellbeing is paramount.

## Author Contributions

Kathleen L. Bagot: conceptualization; data curation; formal analysis; methodology; project administration roles/writing–original draft.

Ryan D. McMullan: data curation; investigation; formal analysis and writing–review and editing.

Johanna I. Westbrook: funding acquisition; methodology; resources and writing–review and editing.

Ling Li: data curation; formal analysis; supervision; validation; and writing–review and editing.

Tim Badgery‐Parker: data curation; formal analysis; visualisation and writing–review and editing.

Rachel Urwin: data curation; investigation; formal analysis and writing–review and editing.

Sandy Middleton: funding acquisition; investigation; methodology; resources and writing–review and editing.

Elizabeth McInnes: conceptualization; funding acquisition; investigation; methodology; resources; supervision and writing–review and editing.

## Funding

This work was supported by the National Health and Medical Research Council Partnership Project Grant (1134459) in partnership with St Vincent’s Health Australia. Open access publishing facilitated by Australian Catholic University, as part of the Wiley ‐ Australian Catholic University agreement via the Council of Australasian University Librarians.

## Disclosure

Funding sources had no involvement in the conduct of the research and/or preparation of the article.

## Conflicts of Interest

The authors declare no conflicts of interest.

## Supporting Information

The Supporting Information has additional information to improve readability and accessibility of information, particularly to assist reader not having to access previously published material regarding data collection methods. Supporting Tables referred to in the manuscript are provided in the Supporting document.

Supporting: Additional information for method and data sources.

Supporting Information Table 1: Demographics of respondents who reported experiencing unprofessional behaviours perpetrated by nurses (Nursing as Perpetrator, LION follow‐up survey, *n* = 782).

Supporting Information Table 2: Frequency of unprofessional behaviours reported about nursing by all professional groups (Nursing as Perpetrator, *Ethos* online reporting system, Reflection Messages, *n* = 538).

Supporting Table 3: Severity class of Reflection messages submitted about nurses (Nurses as Perpetrator, *Ethos* online reporting system, Reflection messages, *n* = 538).

Supporting Information Table 4: Demographics of nursing cohort who completed the LION baseline survey (Nursing as Target or Observer, LION baseline survey, *n* = 2248).

Supporting Information Table 5: Frequency of nursing experiencing incivility/bullying by any professional group (Nursing as Target, LION baseline survey, *n* = 2248).

Supporting Information Table 6: Frequency of nursing experiencing extreme unprofessional behaviour by any professional group (Nursing as Target, LION baseline survey, *n* = 2248).

Supporting Information Table 7: Frequency of 26 unprofessional behaviours experienced by Nursing from any professional group (Nursing as Target, LION baseline survey, *n* = 2248).

Supporting Information Table 8: Frequency of experiencing incivility/bullying by any professional group by role within nursing (Nursing as Target, LION baseline survey, *n* = 2248).

Supporting Information Table 9: Frequency of experiencing extreme unprofessional behaviour by role (Nursing as Target, LION baseline survey, *n* = 2248).

Supporting Information Table 10: Impact on nursing of unprofessional behaviours by any professional group (Nursing as Target, LION baseline survey, *n* = 2248).

Supporting Information Table 11: Frequency of Nursing witnessing incivility/bullying behaviours towards any staff (Nursing as Observer, LION baseline survey, *n* = 2248).

Supporting Information Table 12: Frequency of Nursing witnessing extreme unprofessional behaviour towards any staff (Nursing as Observer, LION baseline survey, *n* = 2248).

Supporting Information Table 13: Frequency of Nursing witnessing incivility/bullying towards any staff by nursing role (Nursing as Observer, LION baseline survey, *n* = 2248).

Supporting Information Table 14: Frequency of Nursing witnessing extreme unprofessional behaviour towards any staff by nursing role (Nursing as Observer, LION baseline survey, *n* = 2248).

Supporting Information Table 15: Agreement by Nurses on statements about speaking up (Nursing as Reporter, LION Follow‐up survey, *n* = 637).

Supporting Information Table 16: Frequency of unprofessional behaviour reported by nursing about professional groups in Reflection messages (Nursing as Reporter, *Ethos* online reporting system, Reflections, *n* = 799).

Supporting Information Table 17: Nursing *Ethos* messengers’ healthcare experience and as an *Ethos* messenger (Nursing as Responder, *Ethos* Messenger survey, *n* = 17).

Supporting Information Table 18: Nursing *Ethos* messenger survey (Nursing as Responder, *Ethos* messenger survey, *n* = 15).

Supporting Information Table 19: Frequency of positive professional behaviours reported by nursing across professional groups (Nursing as Buffer, *Ethos* online reporting system, *n* = 787).

Supporting Information Figure 1: Seniority of staff exhibiting behaviour relative to nursing personnel experiencing behaviour (Nursing as Target, LION follow‐up survey, *n* = 782).

## Supporting information


**Supporting Information** Additional supporting information can be found online in the Supporting Information section.

## Data Availability

The data analysed for the current study are not publicly available due to ethical restrictions.
