# Supplementary material for: A 360‐Degree View of Unprofessional Behaviours Between Nurses and Between Nurses and Medical Colleagues: A Secondary Analysis of a Mixed‐Method Evaluation
Source: J Nurs Manag. 2026 Feb 27;2026:9142351. doi: 10.1155/jonm/9142351 (PMC12949075; doi:10.1155/jonm/9142351)
Supplement: Supplementary file 1 — Supporting Information Additional supporting information can be found online in the Supporting Information section. [file JONM-2026-9142351-s001.docx]

***This document has additional information from the manuscript, to improve readability and accessibility of information, particularly to assist reader not having to access previously published material regarding data collection methods***

**A 360-degree view of unprofessional behaviours between nurses, and between nurses and medical colleagues: a secondary analysis of a mixed-method evaluation**

**Supplemental**

**Method/Data Sources**

Refer to Table 1 in manuscript for summary list of roles in unprofessional behaviour and data source.

Data from the Longitudinal Investigation Of Negative behaviour (LION) Baseline and Followup Surveys, and *Ethos* online system message content were used to examine multiple roles involved in unprofessional behaviour. To do so, each data source was compiled into different data sub-sets specific to the role in unprofessional behaviour being examined. For example, the original data from the *Ethos* online message system reports included reports from all professional groups reporting on all professional groups. This full data set was reduced to examine nurses as perpetrators of unprofessional behaviours by examining reports from all professional groups (participant) reporting specifically on nursing (target). The full data set was also used and reduced differently to examine nurses as reporters of unprofessional behaviours by examining reports only submitted by nurses (participant) reporting on all professional groups (target).

Nursing as Perpetrators of unprofessional behaviour (two data sources)

Data for nurses as perpetrators were sourced from the LION followup survey and the reflection messages submitted to the *Ethos* online reporting system.

Development, administration and results of the all-staff LION baseline and followup surveys are published elsewhere.^1,2^ The on-line survey presented 26 unprofessional staff behaviours (see Table 2 for list) with participants asked to report their experience of each, including frequency within the preceding 12 months and any negative impact on personal wellbeing, teamwork and quality of care. Behaviours were limited to staff, and excluded patient or visitor behaviours. Data for item responses to item stem of ‘*behaviour* has happened to me’ provide behaviour type, frequency (i.e., never, 1-2 times per year, every few months, around monthly, weekly, daily, multiple times daily) and impact (i.e., no impact, minor, moderate, major impact, not sure) for nursing as targets of unprofessional behaviours. Demographics of participants included age-group (i.e., 18-24, 25-34, 35-44, 45-54, 55+, prefer not to answer), gender (i.e., male, female, other, prefer not to answer), nursing role (i.e., enrolled nurse, graduate nurse or midwife, registered nurse or midwife, Nurse Unit Manager [NUM], Associate NUM, clinical nurse consultant, specialist or educator), time employed at current hospital (years) and time employed in sector (years).

The Followup survey included additional items to explore the characteristics of those exhibiting unprofessional behaviours towards nursing. Details captured were the professional group (i.e., Allied/Clinical services, Management/Administrative, Medical, Non-clinical services, Nursing) and seniority (i.e., more junior, same level, more senior). Followup surveys were administered across five hospitals between October 2021 – February 2022 after the implementation of the *Ethos* program. There were 1423 followup surveys completed, with 1247 reporting characteristics of an individual exhibiting unprofessional behaviour, of which 782 were nurses.

The *Ethos* online message system is an anonymous, confidential reporting system available to all staff to report positive (for recognition) and unprofessional (for reflection) behaviours. Input fields include the professional group of the person submitting the report; professional group of the person the report is about; and the date, location and description of the event. These data provide information about the types of unprofessional behaviours experienced or witnessed by whom. Messages are triaged and categorised to one of four levels reflecting severity ranging from ‘level 1 reflect’ to ‘level 4 sanction serious event’ based on message content and if a single or repeated (ongoing pattern) report. Some messages were not able to be categorised due to insufficient information, very minor infringement or if submission considered vexatious. Delivered by trained, peer-level staff (messengers), reflection messages are opportunities for recipients to reflect on their behaviour prior to escalation. Recognition messages provide opportunities to praise specific behaviour and are typically delivered by the recipient’s manager.

Data were deidentified; therefore, the number of unique individuals submitting was not able to be calculated (e.g., one individual may have submitted more than one report). Between July 2017 to December 2020, there were 2504 total submissions from seven hospitals. There were 1310 reflection messages (52.3% of all messages) submitted by clinical (e.g., allied health, medical, nursing) and non-clinical (e.g., administration, food and health services) personnel. Of these, 538 were submitted about nursing’s unprofessional behaviour providing data for nursing as perpetrators.

Nursing as Targets of unprofessional behaviours (two data sources)

Data for nurses as targets of unprofessional behaviours were sourced from the LION Baseline survey completed pre-implementation and the LION Followup survey completed post-implementation of the *Ethos* program intervention.

LION survey details presented in section above, Nursing as Perpetrators. Baseline surveys were administered across eight hospitals between December 2017 and November 2018. Of 5178 completed surveys, 4846 participants reported the experience of at least one unprofessional behaviour, of which 2094 were nurses.

Nursing as Observers of unprofessional behaviours (one data source)

Data for nurses as observers of unprofessional behaviours were sourced from the LION Baseline survey.

The LION baseline survey (detailed in *Nursing as Perpetrators)* included responses to “I have seen this happen to someone else (staff, patients, visitors)”, providing data for nurses as observers of unprofessional behaviours, including type and frequency. Of the 4846 baseline surveys, 2248 were completed by nurses.

Nursing as Reporters of unprofessional behaviours (two data sources)

Data for nurses as reporters were sourced from the LION Followup survey and from reflection messages submitted to the *Ethos* online reporting system.

The LION followup survey included 11 items about speaking up or reporting unprofessional behaviours, including “I have the skills to effectively speak up if I [or others] experience unprofessional behaviour”, “I am encouraged by my colleagues to speak up about unprofessional behaviour” and “I know the proper channels to raise concerns about unprofessional behaviour”. Responses were on a five-point Likert-type scale (strongly disagree to strongly agree). There were 1423 completed followup surveys, of which 691 were completed by nursing.

Of the 1310 reflection messages submitted to the *Ethos* reporting system, 799 were submitted by nursing staff, providing data for nurses as reporters of unprofessional behaviour.

Nursing as Responders to unprofessional behaviours (two data sources)

Data for nursing as responders to unprofessional behaviours were sourced from the middle manager interviews and the *Ethos* Messenger survey.

One-on-one individual interviews were conducted with middle managers from medicine, nursing and non-clinical support services to explore their experiences of unprofessional behaviours. Interview results for all professional groups are previously published,^3^inclduing the interview schedule. Participants were interviewed remotely about unprofessional behaviours they experienced, witnessed or had raised with them by team members. Interviews were conducted between August 2020 and May 2021, taking approximately 60 minutes each (on average), and were recorded and transcribed for analysis. There were 30 middle manager participants, 12 of whom were nurses.

The *Ethos* peer messengers who delivered recognition or reflection messages were invited to complete a 21-item (17 closed, 4 open-ended) survey targeting their experiences in delivering reflection messages. Items covered reasons for becoming an *Ethos* messenger; logistics involved (e.g., training, capacity); rewards and challenges; and views on *Ethos* program. The survey also included items for how long participants had worked in healthcare, employed at the current hospital and how long they had been an *Ethos* messenger. Surveys were administered across eight hospitals, and completed between 16 October and 25 November 2020. Full results are published elsewhere^4^ Of approximately 145 messengers, there were 60 respondents (41.4% response rate), 17 were from nursing (28%).

Nursing as buffers against unprofessional behaviour (two data sources)

Data for nursing as buffering staff against unprofessional behaviour were sourced from the Recognition messages submitted to the *Ethos* online reporting system. Two potential buffering roles were identified: providing positive feedback about others’ positive behaviours or nursing exhibiting/demonstrating positive behaviours. Of the 1194 Recognition messages (47.7% of total 2504 submissions), nurses submitted 787 messages providing data for supporting others and 595 messages were submitted about nurses providing data for nurses’ positive behaviours in the workplace.

**Supplementary Material Table 1: Demographics of respondents who reported experiencing unprofessional behaviours perpetrated by nurses (Nursing as Perpetrator, LION followup survey, n=782)**

| **Demographic characteristic** | **Overall (N=782)** |
| --- | --- |
| **Age group (years)** |  |
| 18-24 | 38 (4.9%) |
| 25-34 | 230 (29.4%) |
| 35-44 | 195 (24.9%) |
| 45-54 | 174 (22.3%) |
| 55+ | 117 (15.0%) |
| Prefer not to answer | 28 (3.6%) |
| **Gender** |  |
| Male | 115 (14.7%) |
| Female | 644 (82.4%) |
| Other | 3 (0.4%) |
| Prefer not to answer | 20 (2.6%) |
| **Time employed at hospital** |  |
| <1 yr | 105 (13.4%) |
| 1-2 yrs | 107 (13.7%) |
| 3-5 yrs | 177 (22.6%) |
| 6-10 yrs | 157 (20.1%) |
| 11-20 yrs | 175 (22.4%) |
| 20+ yrs | 61 (7.8%) |
| **Time employed in sector** |  |
| <1 yr | 27 (3.5%) |
| 1-2 yrs | 47 (6.0%) |
| 3-5 yrs | 109 (13.9%) |
| 6-10 yrs | 168 (21.5%) |
| 11-20 yrs | 194 (24.8%) |
| 20+ yrs | 236 (30.2%) |
| Missing | 1 (0.1%) |
| **Role** |  |
| Nursing | 508 (65.0%) |
| Medical | 37 (4.7%) |
| Allied Health & Clinical Services | 82 (10.5%) |
| Non-clinical Services | 62 (7.9%) |
| Management & Administrative | 92 (11.8%) |
| Missing | 1 (0.1%) |

Note: LION = Longitudinal Investigation of Negative Behaviour

**Supplementary Material Table 2: Frequency of unprofessional behaviours reported about nursing by all professional groups (Nursing as Perpetrator, Ethos online reporting system, Reflection Messages, n=538)**

| **Behaviours reported in Reflection Messages**  **submitted about nursing**  **(n=1067 behaviours)** | **Total N** | |  | **N by Professional Group reporting about Nursing’s unprofessional behaviours** | | | | | | | | | | | | | | |
| --- | --- | --- | --- | --- | --- | --- | --- | --- | --- | --- | --- | --- | --- | --- | --- | --- | --- | --- |
|  |  |  | **%** | **Nursing** | | | | **Medical** | | | **Allied Health** | | **Other**  **Clinical** | | | **Non-Clinical** | | |
|  |  |  | | | **n** | **%** | **n** | | **%** | **n** | | **%** | | **n** | **%** | | **n** | **%** |
| Being spoken to rudely | **288** | **27%** | | | 217 | 75% | 18 | | 6% | 11 | | 4% | | 12 | 4% | | 30 | 10% |
| Being humiliated or ridiculed | **153** | **14%** | | | 123 | 80% | 11 | | 7% | 4 | | 3% | | 8 | 5% | | 7 | 5% |
| Opinions being ignored | **120** | **11%** | | | 95 | 79% | 9 | | 8% | 3 | | 3% | | 3 | 3% | | 10 | 8% |
| Shouted at or being the target of anger | **104** | **10%** | | | 82 | 79% | 6 | | 6% | 5 | | 5% | | 5 | 5% | | 6 | 6% |
| Being given unreasonable workloads / deadlines / tasks | **67** | **6%** | | | 55 | 82% | 3 | | 4% | 1 | | 1% | | 3 | 4% | | 5 | 7% |
| Having unjustified allegations made | **66** | **6%** | | | 55 | 83% | 4 | | 6% | 3 | | 5% | | 2 | 3% | | 2 | 3% |
| Excessive monitoring of work | **48** | **4%** | | | 43 | 90% | 2 | | 4% | 0 | | 0% | | 2 | 4% | | 1 | 2% |
| Someone withholding information which affects work performance | **44** | **4%** | | | 35 | 80% | 3 | | 7% | 3 | | 7% | | 1 | 2% | | 2 | 5% |
| Being ignored or excluded | **33** | **3%** | | | 28 | 85% | 0 | | 0% | 0 | | 0% | | 1 | 3% | | 4 | 12% |
| Repeated reminders of errors or mistakes | **32** | **3%** | | | 28 | 88% | 0 | | 0% | 0 | | 0% | | 4 | 13% | | 0 | 0% |
| Physically intimidating behaviours | **23** | **2%** | | | 21 | 91% | 0 | | 0% | 0 | | 0% | | 1 | 4% | | 1 | 4% |
| Negative comments or offensive jokes -discriminatory | **20** | **2%** | | | 18 | 90% | 0 | | 0% | 1 | | 5% | | 0 | 0% | | 1 | 5% |
| Being the subject of excessive teasing / sarcasm | **18** | **2%** | | | 13 | 72% | 2 | | 11% | 0 | | 0% | | 1 | 6% | | 2 | 11% |
| Treated unfairly – discriminatory | **18** | **2%** | | | 16 | 89% | 0 | | 0% | 1 | | 6% | | 1 | 6% | | 0 | 0% |
| Graphic intrusive comments / questions / insinuations | **8** | **1%** | | | 5 | 63% | 0 | | 0% | 0 | | 0% | | 1 | 13% | | 2 | 25% |
| Having key areas of responsibility removed or replaced with meaningless or unpleasant tasks | **8** | **1%** | | | 8 | 100% | 0 | | 0% | 0 | | 0% | | 0 | 0% | | 0 | 0% |
| Hints or signals from others to quit your job | **6** | **1%** | | | 6 | 100% | 0 | | 0% | 0 | | 0% | | 0 | 0% | | 0 | 0% |
| Physical assault^ | **4** | **0%** | | | 4 | 100% | 0 | | 0% | 0 | | 0% | | 0 | 0% | | 0 | 0% |
| Being told sexually explicit or offensive jokes / comments at work | **2** | **0%** | | | 2 | 100% | 0 | | 0% | 0 | | 0% | | 0 | 0% | | 0 | 0% |
| Inappropriate or unwanted touching^ | **2** | **0%** | | | 2 | 100% | 0 | | 0% | 0 | | 0% | | 0 | 0% | | 0 | 0% |
| Unwelcome sexual flirtations / persistent requests for dates | **1** | **0%** | | | 0 | 0% | 0 | | 0% | 0 | | 0% | | 0 | 0% | | 1 | 100% |
| Being shown sexually suggestive media | **1** | **0%** | | | 1 | 100% | 0 | | 0% | 0 | | 0% | | 0 | 0% | | 0 | 0% |
| Threats of violence / physical abuse^ | **1** | **0%** | | | 1 | 100% | 0 | | 0% | 0 | | 0% | | 0 | 0% | | 0 | 0% |
| Unwelcome practical jokes | **0** |  | | | 0 |  | 0 | |  | 0 | |  | | 0 |  | | 0 |  |
| Demands for sexual favours^ | **0** |  | | | 0 |  | 0 | |  | 0 | |  | | 0 |  | | 0 |  |
| Sexual assault^ | **0** |  | | | 0 |  | 0 | |  | 0 | |  | | 0 |  | | 0 |  |

Note: ^ 5 behaviour types categorised as extreme unprofessional behaviours; remaining 21 categorised as incivility/bullying

**Supplementary Table 3: Severity class of Reflection messages submitted about Nurses
(Nurses as Perpetrator, Ethos online reporting system, Reflection Messages, n=538)**

| **Severity Class** | **N** | **All responses** | **Categorised and actionable responses** |
| --- | --- | --- | --- |
|  |  | **% of 542** | **% of 336** |
| Level 1 Reflect | 197 | 36 | 59 |
| Level 2 Alert Apparent pattern | 67 | 12 | 20 |
| Level 2 Alert Serious event | 47 | 9 | 14 |
| Level 3 Account Notifiable event | 4 | 1 | 1 |
| Level 3 Account Ongoing pattern | 11 | 2 | 3 |
| Level 3 Account Serious event | 10 | 2 | 3 |
| Level 4 Sanction Serious event | 0 | 0 | 0 |
| Not Categorised | 57 | 11 | n/a |
| Unactionable | 149 | 27 | n/a |
| **Total** | **542** | **100** | **100** |

**Supplementary Material Table 4: Demographics of nursing cohort who completed the LION baseline survey (Nursing as Target or Observer, LION baseline survey, n=2248)**

| **Demographic characteristic** | **Overall (N=2248)** |
| --- | --- |
| **Age group (years)** |  |
| 18-24 | 162 (7.2%) |
| 25-34 | 777 (34.6%) |
| 35-44 | 433 (19.3%) |
| 45-54 | 455 (20.2%) |
| 55+ | 367 (16.3%) |
| Prefer not to answer | 53 (2.4%) |
| Missing | 1 (0.0%) |
| **Gender** |  |
| Male | 281 (12.5%) |
| Female | 1926 (85.7%) |
| Other | 2 (0.1%) |
| Prefer not to answer | 37 (1.6%) |
| Missing | 2 (0.1%) |
| **Nursing role** |  |
| Clinical Nurse Consultant/Specialist/Educator | 410 (18.2%) |
| Enrolled Nurse | 150 (6.7%) |
| Graduate Nurse or Midwife | 105 (4.7%) |
| Nurse Unit Manager or Associate NUM | 257 (11.4%) |
| Registered Nurse or Midwife | 1285 (57.2%) |
| Missing | 41 (1.8%) |
| **Time employed at hospital** |  |
| <1 year | 290 (12.9%) |
| 1-2 years | 321 (14.3%) |
| 3-5 years | 489 (21.8%) |
| 6-10 years | 469 (20.9%) |
| 11-20 years | 465 (20.7%) |
| 20+ years | 200 (8.9%) |
| Missing | 14 (0.6%) |
| **Time employed in sector** |  |
| <1 year | 111 (4.9%) |
| 1-2 years | 132 (5.9%) |
| 3-5 years | 318 (14.1%) |
| 6-10 years | 459 (20.4%) |
| 11-20 years | 470 (20.9%) |
| 20+ years | 719 (32.0%) |
| Missing | 39 (1.7%) |

Note: LION = Longitudinal Investigation of Negative Behaviour

**Supplementary Material Table 5: Frequency of nursing experiencing incivility/bullying by any professional group (Nursing as Target, LION baseline survey, n=2248)**

| Experienced incivility/bullying | N | Percentage of non-missing responses | | |
| --- | --- | --- | --- | --- |
|  |  | % | 95%LL | 95%UL |
| Missing | 86 |  |  |  |
| Never | 76 | 3.5 | 1.3 | 5.8 |
| 1-2x Year to Monthly | 1,156 | 53.5 | 51.3 | 55.7 |
| Weekly to Multiple Daily | 930 | 43.0 | 40.8 | 45.3 |

Note: incivility/bullying behaviours include being ignored or excluded, being spoken to rudely, being given unreasonable workload or deadlines, repeated reminders of errors or mistakes, physically intimidating behaviours, etc.). Individual behaviours are reported in Table 7.

**Supplementary Material Table 6: Frequency of nursing experiencing extreme unprofessional behaviour by any professional group (Nursing as Target, LION baseline survey, n=2248)**

| Experienced extreme unprofessional behaviour | N | Percentage of non-missing responses | | |
| --- | --- | --- | --- | --- |
|  |  | % | 95%LL | 95%UL |
| Missing | 15 |  |  |  |
| Never | 1,790 | 80.2 | 78.5 | 81.9 |
| Ever | 443 | 19.8 | 18.2 | 21.5 |

Note: Extreme unprofessional behaviours were physical assault, threats of violence or physical abuse, inappropriate or unwanted touching, demands for sexual favours, sexual assault). Individual behaviours are reported in Table 7. LL=Lower limit, UL=Upper limit.

**Supplementary Material** **Table 7: Frequency of 26 unprofessional behaviours experienced by Nursing from any professional group (Nursing as Target*,* LION baseline survey, n=2248)**

| **Behaviour** | **Frequency** | | | | | | | | | | | | | **Missing#** | |
| --- | --- | --- | --- | --- | --- | --- | --- | --- | --- | --- | --- | --- | --- | --- | --- |
|  | **Never** | | | | **Occasionally**  ***around monthly to 1-2 times a year*** | | | | **Frequently**  ***weekly to multiple times daily*** | | | | |  |  |
|  | **N** | **%** | **LL** | **UL** | **N** | **%** | **LL** | **UL** | **N** | **%** | **LL** | **UL** | **N** | |  |
| Being spoken to rudely | 247 | 11.06 | 9.14 | 13.06 | 1511 | 67.67 | 65.74 | 69.67 | 475 | 21.27 | 19.35 | 23.27 | 15 | |  |
| Someone withholding information which affects work performance | 1053 | 47.58 | 45.37 | 49.84 | 903 | 40.80 | 38.59 | 43.06 | 257 | 11.61 | 9.40 | 13.87 | 35 | |  |
| Opinions being ignored | 516 | 23.14 | 20.99 | 25.31 | 1259 | 56.46 | 54.30 | 58.63 | 455 | 20.40 | 18.25 | 22.57 | 18 | |  |
| Shouted at or being the target of anger | 971 | 43.64 | 41.44 | 45.87 | 1074 | 48.27 | 46.07 | 50.50 | 180 | 8.09 | 5.89 | 10.32 | 23 | |  |
| Being told sexually explicit or offensive jokes / comments at work | 1431 | 64.49 | 62.46 | 66.57 | 682 | 30.73 | 28.71 | 32.82 | 106 | 4.78 | 2.75 | 6.86 | 29 | |  |
| Physically intimidating behaviours | 1483 | 66.41 | 64.44 | 68.47 | 660 | 29.56 | 27.59 | 31.61 | 90 | 4.03 | 2.06 | 6.09 | 15 | |  |
| Hints or signals from others to quit your job | 1828 | 82.12 | 80.59 | 83.72 | 346 | 15.54 | 14.02 | 17.15 | 52 | 2.34 | 0.81 | 3.94 | 22 | |  |
| Repeated reminders of errors or mistakes | 1319 | 59.20 | 57.09 | 61.35 | 793 | 35.59 | 33.48 | 37.75 | 116 | 5.21 | 3.10 | 7.36 | 20 | |  |
| Excessive monitoring of work | 1339 | 59.99 | 57.89 | 62.11 | 663 | 29.70 | 27.60 | 31.82 | 230 | 10.30 | 8.20 | 12.42 | 16 | |  |
| Unwelcome practical jokes | 1827 | 81.71 | 80.14 | 83.31 | 371 | 16.59 | 15.03 | 18.19 | 38 | 1.70 | 0.13 | 3.30 | 12 | |  |
| Being given unreasonable workloads / deadlines / tasks | 754 | 33.89 | 31.69 | 36.18 | 1067 | 47.96 | 45.75 | 50.24 | 404 | 18.16 | 15.96 | 20.45 | 23 | |  |
| Graphic intrusive comments / questions / insinuations | 1789 | 80.08 | 78.47 | 81.75 | 390 | 17.46 | 15.85 | 19.13 | 55 | 2.46 | 0.85 | 4.13 | 14 | |  |
| Being the subject of excessive teasing / sarcasm | 1695 | 75.81 | 74.06 | 77.62 | 468 | 20.93 | 19.19 | 22.74 | 73 | 3.26 | 1.52 | 5.08 | 12 | |  |
| Threats of violence / physical abuse ^ | 2035 | 90.73 | 89.61 | 91.92 | 191 | 8.52 | 7.40 | 9.70 | 17 | 0.76 | 0.00 | 1.95 | 5 | |  |
| Being ignored or excluded | 1123 | 50.36 | 48.16 | 52.58 | 908 | 40.72 | 38.52 | 42.94 | 199 | 8.92 | 6.73 | 11.14 | 18 | |  |
| Inappropriate or unwanted touching ^ | 1988 | 88.87 | 87.62 | 90.14 | 231 | 10.33 | 9.07 | 11.60 | 18 | 0.80 | 0.00 | 2.07 | 11 | |  |
| Unwelcome sexual flirtations / persistent requests for dates | 2136 | 95.14 | 94.34 | 96.03 | 107 | 4.77 | 3.96 | 5.65 | 2 | 0.09 | 0.00 | 0.97 | 3 | |  |
| Being humiliated or ridiculed | 1619 | 72.44 | 70.60 | 74.36 | 559 | 25.01 | 23.18 | 26.93 | 57 | 2.55 | 0.72 | 4.47 | 13 | |  |
| Demands for sexual favours ^ | 2223 | 99.06 | 98.71 | 99.43 | 21 | 0.94 | 0.58 | 1.30 |  |  |  |  | 4 | |  |
| Having unjustified allegations made | 1777 | 79.76 | 78.10 | 81.42 | 424 | 19.03 | 17.37 | 20.69 | 27 | 1.21 | 0.00 | 2.88 | 20 | |  |
| Having key areas of responsibility removed or replaced with meaningless or unpleasant tasks | 1798 | 80.45 | 78.84 | 82.08 | 369 | 16.51 | 14.90 | 18.14 | 68 | 3.04 | 1.43 | 4.68 | 13 | |  |
| Being shown sexually suggestive media | 2130 | 95.13 | 94.33 | 96.00 | 99 | 4.42 | 3.62 | 5.29 | 10 | 0.45 | 0.00 | 1.32 | 9 | |  |
| Sexual assault ^ | 2227 | 99.29 | 99.02 | 99.64 | 15 | 0.67 | 0.40 | 1.02 | 1 | 0.04 | 0.00 | 0.40 | 5 | |  |
| Physical assault ^ | 2107 | 93.98 | 93.09 | 94.95 | 128 | 5.71 | 4.82 | 6.69 | 7 | 0.31 | 0.00 | 1.29 | 6 | |  |
| Negative comments or offensive jokes -discriminatory | 1736 | 77.67 | 75.97 | 79.42 | 449 | 20.09 | 18.39 | 21.84 | 50 | 2.24 | 0.54 | 3.99 | 13 | |  |
| Treated unfairly - discriminatory | 1872 | 84.44 | 83.00 | 85.95 | 313 | 14.12 | 12.67 | 15.63 | 32 | 1.44 | 0.00 | 2.96 | 31 | |  |

# Missing or Prefer not to answer; Note: ^ 5 behaviour types categorised as extreme unprofessional behaviours; remaining 21 categorised as incivility/bullying. LL=Lower limit, UL=Upper limit.

**Supplementary Material Table 8: Frequency of experiencing incivility/bullying by any professional group by role within nursing (Nursing as Target, LION baseline survey, n=2248)**

| **Nursing Role** | **Experienced incivility/bullying#** | **N** | **Percentage of non-missing responses by nursing role** | | |
| --- | --- | --- | --- | --- | --- |
|  |  |  | **%** | **95%LL** | **95%UL** |
| Enrolled Nurse | Missing | 3 |  |  |  |
|  | Never | 9 | 6.1 | 0.0 | 14.7 |
|  | 1-2x Year to Monthly | 69 | 46.9 | 38.8 | 55.5 |
|  | Weekly to Multiple Daily | 69 | 46.9 | 38.8 | 55.5 |
| Graduate Nurse or Midwife | Missing | 1 |  |  |  |
|  | Never | 2 | 1.9 | 0.0 | 11.7 |
|  | 1-2x Year to Monthly | 56 | 53.8 | 44.2 | 63.6 |
|  | Weekly to Multiple Daily | 46 | 44.2 | 34.6 | 54.0 |
| Registered Nurse or Midwife | Missing | 59 |  |  |  |
|  | Never | 42 | 3.4 | 0.6 | 6.4 |
|  | 1-2x Year to Monthly | 655 | 53.4 | 50.6 | 56.4 |
|  | Weekly to Multiple Daily | 529 | 43.1 | 40.3 | 46.2 |
| Nurse Unit Manager (NUM) or Associate NUM | Missing | 9 |  |  |  |
|  | Never | 9 | 3.6 | 0.0 | 10.4 |
|  | 1-2x Year to Monthly | 127 | 51.2 | 45.2 | 58.0 |
|  | Weekly to Multiple Daily | 112 | 45.2 | 39.1 | 52.0 |
| Clinical Nurse Consultant/Specialist /Educator | Missing | 13 |  |  |  |
|  | Never | 11 | 2.8 | 0.0 | 8.0 |
|  | 1-2x Year to Monthly | 234 | 58.9 | 54.2 | 64.1 |
|  | Weekly to Multiple Daily | 152 | 38.3 | 33.5 | 43.5 |
| Missing |  | 1 |  |  |  |
|  | Never | 3 | 7.5 | 0.0 | 22.7 |
|  | 1-2x Year to Monthly | 14 | 37.5 | 22.5 | 52.7 |
|  | Weekly to Multiple Daily | 22 | 55.0 | 40.0 | 70.2 |

Notes: # Incivility/bullying behaviours include being ignored or excluded, being spoken to rudely, being given unreasonable workload or deadlines, repeated reminders of errors or mistakes, physically intimidating behaviours, etc.). Response categories Never; 1-2 times a year, Every few months, Around monthly; Weekly, Daily, Multiple times daily

95% confidence intervals, LL lower limit, UL upper limit.

**Supplementary Material Table 9: Frequency of experiencing extreme unprofessional behaviour by role (Nursing as Target, LION baseline survey, n=2248)**

| **Role within Nursing** | **Experienced extreme unprofessional behaviour** | **N** | **Percentage of non-missing responses by nursing role** | | |
| --- | --- | --- | --- | --- | --- |
|  |  |  | **%** | **95%LL** | **95%UL** |
| Clinical Nurse Consultant/Specialist/Educator | Missing | 2 |  |  |  |
|  | Never | 347 | 85.0 | 81.3 | 88.2 |
|  | Ever | 61 | 15.0 | 11.8 | 18.7 |
| Enrolled Nurse | Missing | 2 |  |  |  |
|  | Never | 119 | 80.4 | 73.3 | 86.0 |
|  | Ever | 29 | 19.6 | 14.0 | 26.7 |
| Graduate Nurse or Midwife | Never | 88 | 83.8 | 75.6 | 89.6 |
|  | Ever | 17 | 16.2 | 10.4 | 24.4 |
| Nurse Unit Manager or Associate NUM | Missing | 2 |  |  |  |
|  | Never | 196 | 76.9 | 71.3 | 81.6 |
|  | Ever | 59 | 23.1 | 18.4 | 28.7 |
| Registered Nurse or Midwife | Missing | 9 |  |  |  |
|  | Never | 1,010 | 79.2 | 76.8 | 81.3 |
|  | Ever | 266 | 20.8 | 18.7 | 23.2 |
| Missing | Never | 30 | 73.2 | 58.1 | 84.3 |
|  | Ever | 11 | 26.8 | 15.7 | 41.9 |

Note: Extreme unprofessional behaviours were physical assault, threats of violence or physical abuse, inappropriate or unwanted touching, demands for sexual favours, sexual assault).

**Supplementary Material Table 10: Impact on nursing of unprofessional behaviours by any professional group (Nursing as Target*,* LION baseline survey*,* n=2248)**

| **Question** | **No impact** | | **Not sure** | | **Negative impact** | | **Missing**  **N** |
| --- | --- | --- | --- | --- | --- | --- | --- |
|  | **N** | **%** | **N** | **%** | **N** | **%** |  |
| You and your wellbeing | 461 | 20.73 | 32 | 1.44 | 1731 | 77.83 | 24 |
| Other staff and their wellbeing | 255 | 11.58 | 157 | 7.13 | 1791 | 81.30 | 45 |
| Patient care | 562 | 25.29 | 95 | 4.28 | 1565 | 70.43 | 26 |
| Teamwork | 294 | 13.18 | 58 | 2.60 | 1878 | 84.22 | 18 |
| Frequency of errors or mistakes | 498 | 22.45 | 110 | 4.96 | 1610 | 72.59 | 30 |
| Quality of service provided at this hospital | 431 | 19.41 | 94 | 4.23 | 1695 | 76.35 | 28 |

Note: LION = Longitudinal Investigation of Negative Behaviour

| 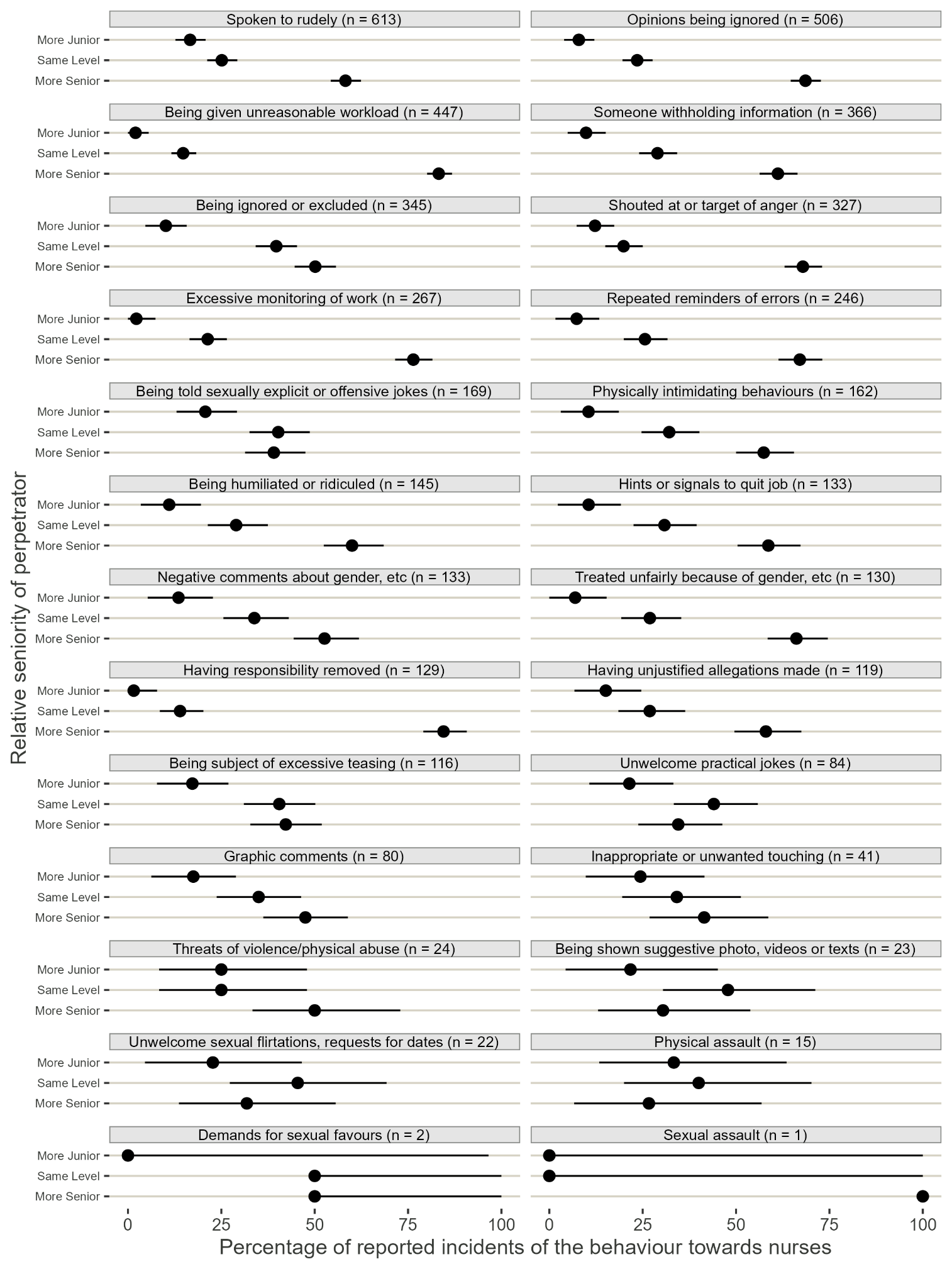  **Supplementary Material Figure 1: Seniority of staff exhibiting behaviour relative to nursing personnel experiencing behaviour (Nursing as Target, LION followup survey, n=782)**  Note: LION = Longitudinal Investigation of Negative Behaviour; Extreme unprofessional behaviours were physical assault, threats of violence or physical abuse, inappropriate or unwanted touching, demands for sexual favours, sexual assault); remaining 21 behaviours categorised as incivility/bullying. |
| --- |

**Supplementary Material Table 11: Frequency of Nursing witnessing incivility/bullying behaviours towards any staff (Nursing as Observer, LION baseline survey, n=2248)**

| Witnessed incivility/bullying# | N | Percentage of non-missing responses | | |
| --- | --- | --- | --- | --- |
|  |  | % | 95%LL | 95%UL |
| Missing | 129 |  |  |  |
| Never | 65 | 3.1 | 0.8 | 5.3 |
| 1-2x Year to Monthly | 973 | 45.9 | 43.7 | 48.2 |
| Weekly to Multiple Daily | 1,081 | 51.0 | 48.8 | 53.3 |

Note: LION = Longitudinal Investigation of Negative Behaviour; # Incivility/bullying behaviours include being ignored or excluded, being spoken to rudely, being given unreasonable workload or deadlines, repeated reminders of errors or mistakes, physically intimidating behaviours, etc.). LL=Lower limit, UL=Upper limit

**Supplementary Material Table 12: Frequency of Nursing witnessing extreme unprofessional behaviour towards any staff (Nursing as Observer, LION baseline survey, n=2248)**

| Witnessed extreme unprofessional behaviour# | N | Percentage of non-missing responses | | |
| --- | --- | --- | --- | --- |
|  |  | % | 95%LL | 95%UL |
| Missing | 29 |  |  |  |
| Never | 1,633 | 73.6 | 71.7 | 75.5 |
| Ever | 586 | 26.4 | 24.6 | 28.3 |

Note: LION = Longitudinal Investigation of Negative Behaviour; #Extreme unprofessional behaviours were physical assault, threats of violence or physical abuse, inappropriate or unwanted touching, demands for sexual favours, sexual assault). LL=Lower limit, UL=Upper limit.

**Supplementary Material Table 13: Frequency of Nursing witnessing incivility/bullying towards any staff by nursing role (Nursing as Observer, LION baseline survey, n=2248)**

| **Role within Nursing** | **Witnessed incivility/bullying** | **N** | **Percentage of non-missing responses by Nursing role** | | |
| --- | --- | --- | --- | --- | --- |
|  |  |  | **%** | **95%LL** | **95%UL** |
| Clinical Nurse Consultant/Specialist/Educator | Missing | 18 |  |  |  |
|  | Never | 4 | 1.0 | 0.0 | 6.2 |
|  | 1-2x Year to Monthly | 190 | 48.5 | 43.4 | 53.6 |
|  | Weekly to Multiple Daily | 198 | 50.5 | 45.4 | 55.7 |
| Enrolled Nurse | Missing | 8 |  |  |  |
|  | Never | 10 | 7.0 | 0.0 | 15.9 |
|  | 1-2x Year to Monthly | 52 | 36.6 | 28.9 | 45.5 |
|  | Weekly to Multiple Daily | 80 | 56.3 | 48.6 | 65.2 |
| Graduate Nurse or Midwife | Missing | 5 |  |  |  |
|  | Never | 3 | 3.0 | 0.0 | 13.8 |
|  | 1-2x Year to Monthly | 44 | 44.0 | 35.0 | 54.8 |
|  | Weekly to Multiple Daily | 53 | 53.0 | 44.0 | 63.8 |
| Nurse Unit Manager or Associate NUM | Missing | 14 |  |  |  |
|  | Never | 4 | 1.6 | 0.0 | 8.4 |
|  | 1-2x Year to Monthly | 115 | 47.3 | 41.2 | 54.1 |
|  | Weekly to Multiple Daily | 124 | 51.0 | 44.9 | 57.8 |
| Registered Nurse or Midwife | Missing | 84 |  |  |  |
|  | Never | 42 | 3.5 | 0.6 | 6.5 |
|  | 1-2x Year to Monthly | 556 | 46.3 | 43.4 | 49.3 |
|  | Weekly to Multiple Daily | 603 | 50.2 | 47.3 | 53.3 |
| Missing | Never | 2 | 4.9 | 0.0 | 20.0 |
|  | 1-2x Year to Monthly | 16 | 39.0 | 24.4 | 54.1 |
|  | Weekly to Multiple Daily | 23 | 56.1 | 41.5 | 71.2 |

Note: # Incivility/bullying behaviours include being ignored or excluded, being spoken to rudely, being given unreasonable workload or deadlines, repeated reminders of errors or mistakes, physically intimidating behaviours, etc.). LL=Lower limit, UL=Upper limit

| **Supplementary Material Table 14: Frequency of Nursing witnessing extreme unprofessional behaviour towards any staff by nursing role (Nursing as Observer, LION baseline survey, n=2248)**   \| **Role within Nursing** \| **Witnessed extreme unprofessional behaviour #** \| **N** \| **Percentage of non-missing responses by Nursing role** \| \| \| \| --- \| --- \| --- \| --- \| --- \| --- \| \| **%** \| **95%LL** \| **95%UL** \| \| Clinical Nurse Consultant/Specialist/Educator \| Missing \| 3 \|  \|  \|  \| \| Never \| 306 \| 75.2 \| 70.8 \| 79.1 \| \| Ever \| 101 \| 24.8 \| 20.9 \| 29.2 \| \| Enrolled Nurse \| Missing \| 1 \|  \|  \|  \| \| Never \| 114 \| 76.5 \| 69.1 \| 82.6 \| \| Ever \| 35 \| 23.5 \| 17.4 \| 30.9 \| \| Graduate Nurse or Midwife \| Missing \| 1 \|  \|  \|  \| \| Never \| 80 \| 76.9 \| 68.0 \| 84.0 \| \| Ever \| 24 \| 23.1 \| 16.0 \| 32.0 \| \| Nurse Unit Manager or Associate NUM \| Missing \| 1 \|  \|  \|  \| \| Never \| 174 \| 68.0 \| 62.0 \| 73.4 \| \| Ever \| 82 \| 32.0 \| 26.6 \| 38.0 \| \| Registered Nurse or Midwife \| Missing \| 23 \|  \|  \|  \| \| Never \| 930 \| 73.7 \| 71.2 \| 76.0 \| \| Ever \| 332 \| 26.3 \| 24.0 \| 28.8 \| \| Missing \| Never \| 29 \| 70.7 \| 55.5 \| 82.4 \| \|  \| Ever \| 12 \| 29.3 \| 17.6 \| 44.5 \| |
| --- | --- | --- | --- | --- | --- | --- | --- | --- | --- | --- | --- | --- | --- | --- | --- | --- | --- | --- | --- | --- | --- | --- | --- | --- | --- | --- | --- | --- | --- | --- | --- | --- | --- | --- | --- | --- | --- | --- | --- | --- | --- | --- | --- | --- | --- | --- | --- | --- | --- | --- | --- | --- | --- | --- | --- | --- | --- | --- | --- | --- | --- | --- | --- | --- | --- | --- | --- | --- | --- | --- | --- | --- | --- | --- | --- | --- | --- | --- | --- | --- | --- | --- | --- | --- | --- | --- | --- | --- | --- | --- | --- | --- | --- | --- | --- | --- | --- | --- | --- | --- | --- |

Note: #Extreme unprofessional behaviours were physical assault, threats of violence or physical abuse, inappropriate or unwanted touching, demands for sexual favours, sexual assault). LL=Lower limit, UL=Upper limit.

**Supplementary Material Table 15: Agreement by Nurses on statements about speaking up (Nursing as Reporter, LION Followup Survey, n=637)**

| **Survey Item** | **Disagree** | |  | | **Neutral** | |  | | **Agree** | |  | | **N/A** | |
| --- | --- | --- | --- | --- | --- | --- | --- | --- | --- | --- | --- | --- | --- | --- |
|  | **N** | | **%** | | **N** | | **%** | | **N** | | **%** | | N | |
| Speaking up or reporting unprofessional behaviour is important for patient safety | 21 | 3.52 | | 22 | | 3.69 | | 553 | | 92.79 | | 41 | |  |
| I am encouraged by my colleagues to speak up about unprofessional behaviour | 91 | 15.35 | | 93 | | 15.68 | | 409 | | 68.97 | | 44 | |  |
| I have the skills to effectively speak up if I experience unprofessional behaviour | 46 | 7.74 | | 87 | | 14.65 | | 461 | | 77.61 | | 43 | |  |
| I have the skills to effectively speak up if others experience unprofessional behaviour | 39 | 6.57 | | 73 | | 12.29 | | 482 | | 81.14 | | 43 | |  |
| I know the proper channels to raise concerns about unprofessional behaviour | 40 | 6.70 | | 51 | | 8.54 | | 506 | | 84.76 | | 40 | |  |
| Unprofessional behaviour is effectively managed in this hospital | 212 | 35.99 | | 180 | | 30.56 | | 197 | | 33.45 | | 48 | |  |
| I feel comfortable speaking up or reporting unprofessional behaviour | 160 | 26.94 | | 111 | | 18.69 | | 323 | | 54.38 | | 43 | |  |
| It takes too much time and effort to report unprofessional behaviour | 194 | 32.72 | | 185 | | 31.20 | | 214 | | 36.09 | | 44 | |  |
| I am confident I would receive support from my supervisor if I reported unprofessional behaviour | 148 | 24.92 | | 112 | | 18.86 | | 334 | | 56.23 | | 43 | |  |
| Speaking up or reporting unprofessional behaviour is likely to have a negative impact on my career | 180 | 30.56 | | 178 | | 30.22 | | 231 | | 39.22 | | 48 | |  |
| I am confident I would be believed and taken seriously if I reported unprofessional behaviour | 103 | 17.43 | | 153 | | 25.89 | | 335 | | 56.68 | | 46 | |  |

**Supplementary Material Table 16: Frequency of unprofessional behaviour reported by nursing about professional groups in Reflection messages (Nursing as Reporter, Ethos online reporting system, Reflections, n=799)**

| **Behaviour Reported in Reflection Messages by Nursing (n=1583 behaviours from 799 Reflection messages)** |  |  | **N by Professional Group reporting about Nursing’s unprofessional behaviours** | | | | | | | | | |
| --- | --- | --- | --- | --- | --- | --- | --- | --- | --- | --- | --- | --- |
|  | **Total** | | Nursing | | Medical | | Allied Health | | Other Clinical | | Non-Clinical | |
|  | **N** | **%** | n | % | n | % | n | % | n | % | n | % |
| Being spoken to rudely | **425** | **27%** | 217 | 51% | 136 | 32% | 8 | 2% | 17 | 4% | 47 | 11% |
| Being humiliated or ridiculed | **217** | **14%** | 123 | 57% | 69 | 32% | 4 | 2% | 8 | 4% | 13 | 6% |
| Opinions being ignored | **195** | **12%** | 95 | 49% | 75 | 38% | 7 | 4% | 4 | 2% | 14 | 7% |
| Shouted at or being the target of anger | **165** | **10%** | 82 | 50% | 64 | 39% | 1 | 1% | 3 | 2% | 15 | 9% |
| Being given unreasonable workloads / deadlines / tasks | **88** | **6%** | 55 | 63% | 21 | 24% | 1 | 1% | 5 | 6% | 6 | 7% |
| Having unjustified allegations made | **88** | **6%** | 55 | 63% | 22 | 25% | 2 | 2% | 4 | 5% | 5 | 6% |
| Someone withholding information which affects work performance | **85** | **5%** | 35 | 41% | 36 | 42% | 3 | 4% | 5 | 6% | 6 | 7% |
| Excessive monitoring of work | **58** | **4%** | 43 | 74% | 10 | 17% | 1 | 2% | 0 | 0% | 4 | 7% |
| Physically intimidating behaviours | **54** | **3%** | 21 | 39% | 14 | 26% | 5 | 9% | 1 | 2% | 13 | 24% |
| Repeated reminders of errors or mistakes | **48** | **3%** | 28 | 58% | 16 | 33% | 0 | 0% | 2 | 4% | 2 | 4% |
| Being ignored or excluded | **36** | **2%** | 28 | 78% | 6 | 17% | 1 | 3% | 1 | 3% | 0 | 0% |
| Being the subject of excessive teasing / sarcasm | **27** | **2%** | 13 | 48% | 9 | 33% | 0 | 0% | 3 | 11% | 2 | 7% |
| Negative comments or offensive jokes -discriminatory | **25** | **2%** | 18 | 72% | 5 | 20% | 1 | 4% | 0 | 0% | 1 | 4% |
| Treated unfairly - discriminatory | **23** | **1%** | 16 | 70% | 4 | 17% | 1 | 4% | 0 | 0% | 2 | 9% |
| Having key areas of responsibility removed or replaced with meaningless or unpleasant tasks | **12** | **1%** | 8 | 67% | 2 | 17% | 0 | 0% | 1 | 8% | 1 | 8% |
| Physical assault ^ | **8** | **1%** | 4 | 50% | 3 | 38% | 0 | 0% | 0 | 0% | 1 | 13% |
| Hints or signals from others to quit your job | **7** | **0%** | 6 | 86% | 1 | 14% | 0 | 0% | 0 | 0% | 0 | 0% |
| Graphic intrusive comments / questions / insinuations | **7** | **0%** | 5 | 71% | 1 | 14% | 0 | 0% | 0 | 0% | 1 | 14% |
| Being told sexually explicit or offensive jokes / comments at work | **5** | **0%** | 2 | 40% | 2 | 40% | 0 | 0% | 0 | 0% | 1 | 20% |
| Inappropriate or unwanted touching ^ | **5** | **0%** | 2 | 40% | 2 | 40% | 0 | 0% | 0 | 0% | 1 | 20% |
| Threats of violence / physical abuse ^ | **3** | **0%** | 1 | 33% | 2 | 67% | 0 | 0% | 0 | 0% | 0 | 0% |
| Being shown sexually suggestive media | **1** | **0%** | 1 | 100% | 0 | 0% | 0 | 0% | 0 | 0% | 0 | 0% |
| Sexual assault ^ | **1** | **0%** | 0 | 0% | 1 | 100% | 0 | 0% | 0 | 0% | 0 | 0% |
| Unwelcome practical jokes | **0** |  | 0 |  | 0 |  | 0 |  | 0 |  | 0 |  |
| Unwelcome sexual flirtations / persistent requests for dates | **0** |  | 0 |  | 0 |  | 0 |  | 0 |  | 0 |  |
| Demands for sexual favours ^ | **0** |  | 0 |  | 0 |  | 0 |  | 0 |  | 0 |  |
|  | **1583** | **100%** | 858 | 54% | 501 | 32% | 35 | 2% | 54 | 3% | 135 | 9% |

Note: ^ 5 behaviour types categorised as extreme unprofessional behaviours; remaining 21 categorised as incivility/bullying

**Supplementary Material Table 17: Nursing Ethos Messengers’ healthcare experience and as an Ethos Messenger (Nursing as Responder, Ethos Messenger survey, n=17)**

| **Healthcare experience** | | | |  | | **Ethos Messenger** | | |
| --- | --- | --- | --- | --- | --- | --- | --- | --- |
| **Years** | **How long employed at hospital**  **n, %** | **How long worked in healthcare**  **n, %** |  | | **Months** | | **How long an Ethos Messenger**  **n, %** |  |
| Less than 1 year -2 years | 0 | 0 |  | | <3 months | | 1, 6% |  |
| 3-5 years | 4, 24% | 1, 6% |  | | 3-6 months | | 3, 18% |  |
| 6-10 years | 7, 41% | 6, 35% |  | | 6-12 months | | 4, 24% |  |
| 11-20 years | 2, 12% | 3, 18% |  | | >12 months | | 7, 41% |  |
| Over 20 years | 4, 24% | 7, 41% |  | | Missing | | 2, 12% |  |

Nearly all (16, 94%) had worked in health care for more than 5 years with nearly half (n=7, 41%) as an Ethos Messenger for over twelve months.

**Supplementary Material Table 18: Nursing Ethos Messenger survey (Nursing as Responder, Ethos Messenger survey, n=15)**

| **Item** | **Strongly agree**  **n,%** | **Somewhat agree**  **n,%** | **Neither agree nor disagree n,%** | **Somewhat disagree n,%** | **Strongly disagree n,%** | **Missing**  **n,%** |
| --- | --- | --- | --- | --- | --- | --- |
| My responsibilities as an Ethos messenger are clearly defined | 9, 60% | 4, 27% | 1, 7% | 1, 7% | - | - |
| I have access to the support I need to fulfill my Ethos messenger role | 10, 67% | 3, 20% | 1, 7% | 1, 7% | - | - |
| I have the skills needed to carry out the Ethos messenger role successfully | 8, 53% | 6, 40% | - | 1, 7% | - | - |
| I am satisfied with the Ethos messenger training I have received | 9, 60% | 4, 27% | 2, 13% | - | - | - |
| Hospital management is committed to the success of the Ethos program | 6, 40% | 3, 20% | 3, 20% | 3, 20% | - | - |
| The Ethos message triage system is effective in ensuring the feedback I am asked to deliver is appropriate and meaningful | 5, 33% | 5, 33% | 2, 13% | 1, 7% | - | 2, 13% |
| I have enough time to carry out the Ethos messenger role effectively | 3, 20% | 5, 33% | 2, 13% | 3, 20% | - | 2, 13% |
| My role as an Ethos messenger has negatively impacted my relationship with colleagues/other hospital staff | - | - | 5, 33% | 3, 20% | 5, 33% | 2, 13% |
| There is sufficient follow-up and support available for recipients of Ethos messages | 6, 40% | 1, 7% | 3, 20% | 3, 20% | - | 2, 13% |
| I am satisfied with the way the Ethos program is being managed at my hospital | 6, 40% | 5, 33% | 1, 7% | 3, 20% | - | - |
| Being an Ethos messenger has increased my awareness of unprofessional behaviours in the workplace | 7, 47% | 6, 40% | 1, 7% | - | 1, 7% | - |
| The Ethos program will lead to a decrease in unprofessional behaviour in my hospital | 3, 20% | 7, 47% | 4, 27% | 1, 7% | - | - |
| I would recommend being an Ethos messenger to colleagues | 8, 53% | 3, 20% | 3, 20% | 1, 7% | - | - |
| I feel valued for being an Ethos messenger | 6, 40% | 2, 13% | 5, 33% | 2, 13% | - | - |

**Supplementary Material Table 19: Frequency of positive professional behaviours reported by Nursing across professional groups (Nursing as Buffer, Ethos online reporting system, n=787)**

| **Behaviour Reported in Recognition Messages BY Nursing**  **(n=1930 positive behaviours reported)** | **N by Professional Group Nursing reporting positive behaviours about** | | | | | | | | | | | |
| --- | --- | --- | --- | --- | --- | --- | --- | --- | --- | --- | --- | --- |
|  | **Total** |  | Nursing | | Medical | | Allied Health | | Other Clinical | | Non-Clinical | |
|  |  | **%** | n | % | n | % | n | % | n | % | n | % |
| Patient care delivery and performance enhanced (treatment and care effectiveness, safety, manner and quality of care, patient-centredness and timeliness) | **425** | **22%** | 269 | 63% | 94 | 22% | 13 | 3% | 18 | 4% | 31 | 7% |
| Notable non-technical skills demonstrated (teamwork, coordination, collaboration, communication, other) | **625** | **32%** | 385 | 62% | 105 | 17% | 17 | 3% | 30 | 5% | 88 | 14% |
| Notable technical skills demonstrated | **22** | **1%** | 9 | 41% | 3 | 14% | 0 | 0% | 2 | 9% | 8 | 36% |
| Overcompensating for gaps in resources, task, skill shortages through personal effort and time | **61** | **3%** | 40 | 66% | 8 | 13% | 1 | 2% | 4 | 7% | 8 | 13% |
| Positive values-driven behaviour demonstrated (ethical, compassionate, empathetic, supportive, respectful, affording others dignity) | **579** | **30%** | 370 | 64% | 93 | 16% | 13 | 2% | 27 | 5% | 76 | 13% |
| Advocacy for other people demonstrated | **41** | **2%** | 27 | 66% | 8 | 20% | 3 | 7% | 2 | 5% | 1 | 2% |
| Positive leadership, teaching or coaching skills demonstrated | **177** | **9%** | 135 | 76% | 28 | 16% | 1 | 1% | 7 | 4% | 6 | 3% |
| Total | **1930** | **100%** | 1235 | 64% | 339 | 18% | 48 | 2% | 90 | 5% | 218 | 11% |
